# Biological Features and In Planta Transcriptomic Analyses of a *Microviridae* Phage (CLasMV1) in “*Candidatus* Liberibacter asiaticus”

**DOI:** 10.3390/ijms231710024

**Published:** 2022-09-02

**Authors:** Cheng Wang, Fang Fang, Yun Li, Ling Zhang, Jinghua Wu, Tao Li, Yongqin Zheng, Qian Xu, Shuting Fan, Jianchi Chen, Xiaoling Deng, Zheng Zheng

**Affiliations:** 1 Guangdong Province Key Laboratory of Microbial Signals and Disease Control, South China Agricultural University, Guangzhou 510642, China; 2 San Joaquin Valley Agricultural Sciences Center, Agricultural Research Service, United States Department of Agriculture, Parlier, CA 90089, USA

**Keywords:** *Microviridae*, “*Candidatus* Liberibacter asiaticus”, population dynamics, distribution, transcriptome, interaction

## Abstract

“*Candidatus* Liberibacter asiaticus” (CLas) is the causal agent of citrus Huanglongbing (HLB, also called citrus greening disease), a highly destructive disease threatening citrus production worldwide. A novel *Microviridae* phage (named CLasMV1) has been found to infect CLas, providing a potential therapeutic strategy for CLas/HLB control. However, little is known about the CLasMV1 biology. In this study, we analyzed the population dynamics of CLasMV1 between the insect vector of CLas, the Asian citrus psyllid (ACP, *Diaphorina citri* Kuwayama) and the holoparasitic dodder plant (*Cuscuta campestris* Yunck.); both acquired CLasMV1-infected CLas from an HLB citrus. All CLas-positive dodder samples were CLasMV1-positive, whereas only 32% of CLas-positive ACP samples were identified as CLasMV1-positive. Quantitative analyses showed a similar distribution pattern of CLasMV1 phage and CLas among eight citrus cultivars by presenting at highest abundance in the fruit pith and/or the center axis of the fruit. Transcriptome analyses revealed the possible lytic activity of CLasMV1 on CLas in fruit pith as evidenced by high-level expressions of CLasMV1 genes, and CLas genes related to cell wall biogenesis and remodeling to maintain the CLas cell envelope integrity. The up-regulation of CLas genes were involved in restriction–modification system that could involve possible phage resistance for CLas during CLasMV1 infection. In addition, the regulation of CLas genes involved in cell surface components and Sec pathway by CLasMV1 phage could be beneficial for phage infection. This study expanded our knowledge of CLasMV1 phage that will benefit further CLas phage research and HLB control.

## 1. Introduction

“*Candidatus* Liberibacter asiaticus” (CLas) is the causal agent of citrus Huanglongbing (HLB), the most destructive disease in citrus production worldwide. CLas is a phloem-limited α-proteobacterium which currently has not yet been cultured in vitro, which leads to the CLas characterization being mainly based on the analysis of infected host tissue. CLas is transmitted between citrus plants in the field mainly by the phloem-feeding Asian citrus psyllid (ACP, *Diaphorina citri* Kuwayama) [1]. Experimentally or for scientific research, CLas can be transmitted via dodder (*Cuscuta campestris* Yunck.), a holoparasitic plant, from one infected citrus plant to other citrus plants or other amenable hosts, e.g., periwinkle (*Catharanthus roseus* (L.) G. Don) [2]. CLas can infect most commercial citrus cultivars and present as an uneven distribution pattern within infected citrus plants [3,4]. The distribution pattern of CLas in infected citrus trees could be different, mainly due to the different cultivars and degrees of disease severity [5,6]. However, an investigation of CLas distribution in 14 commercial citrus cultivars showed that a significantly high abundance of CLas was stably observed in fruit pith tissue compared to other tissues from the individual citrus branch [4].

Advances in high-throughput sequencing technology have extended the understanding of CLas biology, especially the discoveries of CLas phages (viruses that infect CLas) [7,8,9,10]. Currently, four types of CLas phages/prophages have been identified, including Type 1 (SC1), Type 2 (SC2), Type 3 (P-JXGC-3) and a *Microviridae* phage (CLasMV1) [7,8,10]. SC1 carried putative lytic cycle genes and was found in lytic form only in planta [7]. SC2 was found to lack lytic cycle genes and found to be involved in lysogenic conversion [7]. P-JXGC-3 also lacked the lytic capacity and carried a restriction–modification system (RM system), which could protect CLas against other forms of phage invasion [8]. Variant forms of Type 1 and Type 2 prophage have been reported in CLas strains from Pakistan [11]. CLasMV1, a single-stranded DNA phage with a circular genome (~8.8 kb) was recently identified and found to be widely distributed in the CLas population in China [10].

The discovery of CLas phages/prophages has opened a new venue for CLas biology research. In particular, the prophage, an integrated form of phage genome in the CLas genome, has played critical roles in the genomic evolution, pathogenicity, adaptability and survival of CLas [8,12,13,14,15,16,17]. A peroxidase gene (SC2_gp095) encoded by Type 2 prophage (SC2) was found to be involved in the fitness and survival of CLas in the host plant by the suppression of the reactive oxygen-mediated host defenses [13]. Two late genes (holin, SC1_gp110 and endolysins, SC1_gp035) of Type 1 prophage (SC1) showed an inhibition of bacterial growth by expression in *Escherichia coli*, suggesting that their function could be involved in limiting CLas cultivability [12]. Transgenic citrus expressing a prophage-encoded protein (LasP235) showed the HLB-like symptoms and altered biosynthesis of secondary metabolites pathways in Carrizo citrange (*Citrus sinensis* × *Poncirus trifoliata*) [18]. In a recent study, the transient expression of a nonclassical secretory prophage-encoded protein (AGH17470) caused the hypersensitive response and dwarfing of *Nicotiana benthamiana* Domin, and also up-regulated the pathogenesis-related genes and promoted SA accumulation in citrus plants, indicating its important role in influencing plant immune response and growth [17]. Unlike SC1, SC2 and P-JXGC-3, which contained a large genome (>30 kb) with a prophage form [8], the prophage form of CLasMV1 has not been found [10]. However, as a widely distributed phage in the CLas population in China [10], the biology of CLasMV1 phage remains unclear.

In this study, the population dynamics of CLasMV1 phage in CLas samples from three hosts (ACP, dodder and citrus) was analyzed. We also investigated the genome-wide gene expression of CLasMV1 phage and CLas in CLas-infected plant tissue using dual-RNA sequencing. The RNA-Seq data provided the evidence of the lytic activity of CLasMV1 in CLas-infected fruit pith samples. Comparative transcriptomic analyses of CLas samples that contained high-abundance and low-abundance CLasMV1 were performed to reveal how CLas responds to CLasMV1 infection. Our results not only expanded the understanding of the *Microviridae* phage activity, but also provided new insights into the CLas-phage interaction.

## 2. Results

### 2.1. Acquisition and Population Dymatic of CLasMV1 and CLas in Psyllid

For ACP assay, all 40 psyllids showed as CLas-positive after two weeks’ feeding on CLas-infected citrus plants (Table 1). Although all citrus plants used for ACP feeding were CLasMV1-positive, only 13 CLas-infected psyllids (32.5%) showed positive for CLasMV1 (Table 1). The number of CLasMV1-positive psyllids that were raised on citrus plants varied among plants, ranging from two to five (Table 1). Quantification analyses showed that CLasMV1 did not replicate to a high level in ACP with the maximum of 4.77 copies per CLas cell, compared to the maximum of 58.20 copies per CLas cell in the host citrus (Table 1). The acquisition ratio of CLasMV1 by psyllid was not correlated with the density of CLasMV1 phage in the host plant. For instance, both citrus plant #CT1 and #CT2 contained a high amount of CLasMV1 (>20 copies of CLasMV1 phage per CLas cell), while only two out of ten psyllids showed positive after feeding on plant #CT1 or #CT2 (Table 1). In contrast, five out of ten psyllids were identified as CLasMV1-positive after two weeks’ feeding on citrus plant #CT3, which only contained ~1.2 copies of CLasMV1 phage per CLas cell (Table 1).

### 2.2. Acquisition and Population Dymatic of CLasMV1 and CLas in Dodder

For dodder assay, a total of 20 CLas-infected citrus branches that showed CLasMV1-positive (Ct value ranging from 15.92 to 26.13) were used as host sources for dodders’ parasitizing. All dodders grown on 20 citrus branches were infected by CLas with Ct value ranging from 15.68 to 26.43 after 14 days’ parasitizing (Table 2). CLasMV1 was detected in all 20 dodder tendrils with Ct value ranging from 11.55 to 25.15, indicating the stable acquisition of CLasMV1 phage by dodders. Quantification analyses showed a higher density (not significant) of CLasMV1 was observed in dodder tendrils (average of 19.39 copies per CLas cell) compared to those in the parasitized citrus branch (average of 12.97 copies per CLas cell) (Table 2).

**Figure 1:**
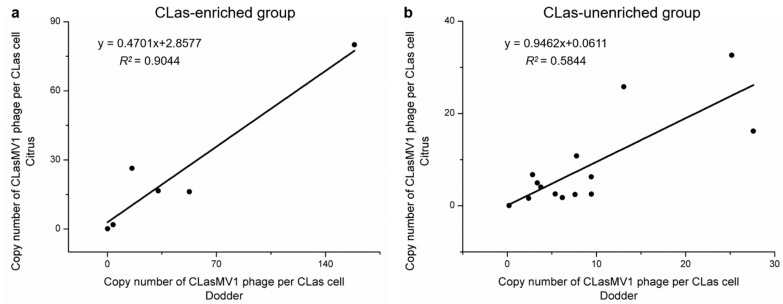
Linear trend line analysis of CLasMV1 phage density in dodder and in the parasitizing citrus branch. (**a**) The CLas-enriched group; (**b**) CLas-unenriched group.

Compared to the corresponding citrus branches, a higher CLas concentration was observed in six (i.e., CLas-enriched dodder group) out of twenty dodders by showing the lower Ct value of CLas in dodder tendril (Table 2). Notably, the density of CLasMV1 phage in CLas-enriched dodder tendrils was highly correlated (*R*^2^ = 0.9044) with the density of CLasMV1 in the corresponding citrus branch (Figure 1). However, for the dodder tendril (i.e., CLas-unenriched dodder group) that contained lower CLas concentration than the parasitizing citrus branch, a lower correlation (*R*^2^ = 0.5844) was observed between the density of CLasMV1 phage in the dodder tendril and in the corresponding citrus branch (Figure 1).

### 2.3. Quantitative Distribution of CLasMV1 Phage in CLas-Infected Citrus Branch

**Figure 2:**
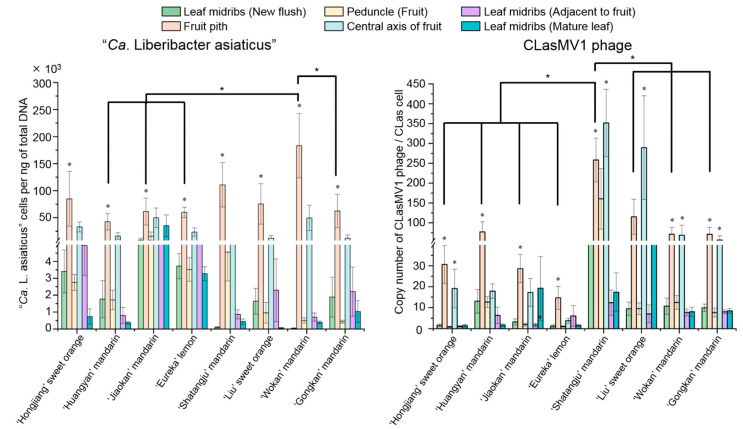
Quantification analyses of “*Candidatus* Liberibacter asiaticus” and CLasMV1 phage in six types of tissues from citrus branch. Single factor ANOVA (Duncan’s multiple range test) at 95% (*p* < 0.05) confidence interval is used to determine statistical significance. *: *p* < 0.05.

The distribution of CLasMV1 phage in citrus was analyzed in six different types of tissue from the individual CLas-infected branch of eight citrus cultivars (Figure S1). Quantification analyses revealed that CLasMV1 phage was most abundant in the fruit pith and/or the center axis of the CLas-infected citrus branch from eight cultivars, which was similar to the distribution pattern of CLas in the citrus branch by presenting at the highest population in the fruit pith tissue (Figure 2). However, the population of CLasMV1 and CLas in fruit pith varied among citrus cultivars and showed a different abundance pattern (Figure 2). The highest abundance of CLasMV1 was observed in the fruit pith of ‘Shatangju’ mandarin (~258 copies/CLas cell), significantly different from other seven cultivars (*p* < 0.05). However, the highest concentration of CLas was detected in the fruit pith tissue of ‘Wokan’ mandarin, significantly higher than those of ‘Huangyan’ mandarin, ‘Jiaokan’ mandarin, ‘Gongkan’ mandarin and ‘Eureka’ lemon (*p* < 0.05) (Figure 2). Among eight cultivars, the lowest density of CLasMV1 phage in the fruit pith was observed in ‘Eureka’ lemon, while CLas showed the lowest concentration in the fruit pith of ‘Huangyan’ mandarin (Figure 2).

### 2.4. Genome-Wide Transcriptome Analyses of CLasMV1 Phage and CLas in Leaf Midribs and Fruit Pith

To characterize the gene expression pattern of CLasMV1 and analyses of CLas gene response during CLasMV1 infection, CLas samples with low-abundant CLasMV1 (leaf midribs) and high-abundant CLasMV1 (fruit pith) were selected for dual RNA-Seq and transcriptomic analyses. The average density of CLasMV1 in three fruit pith samples was about 347.86 copies per CLas cell, in comparison to the average of 4.18 copies per CLas cell observed in leaf samples (Table 3). Approximately 381 million and 408 million HiSeq reads were generated from CLas-infected leaf midribs and fruit pith RNA samples, respectively. Read mapping to CLas genomes (A4 strain, CP010804.2) and CLasMV1 genome (CP045566.1) identified a total of 822,078 reads from fruit pith RNA-Seq data and 67,354 reads from leaf midribs RNA-Seq data (Table 3). A total of 50,829 reads (6.18% of total mapped reads) were identified as CLasMV1 reads in fruit pith RNA-Seq data, while only 366 CLasMV1 reads (0.54% of total mapped reads) were identified in leaf midribs RNA-Seq data (Table 3). The normalizing of RNA-seq data with TPM showed that the expression level of CLasMV1 genes (with average TPM value = 8303) was relatively higher than the expression level of CLas genes (with average TPM value = 875) in fruit pith tissue (Table 4, Supplementary Table S1). In particular, GE519_gp08, a major capsid protein (MCP), showed a high expression, with TPM values of 9880 (Table 4). In contrast, the expression level of CLasMV1 genes (with average TPM value = 787) was slightly lower than those of CLas genes in leaf midribs tissue (with average TPM value = 931) (Table 4, Supplementary Table S1). Compared to leaf midribs tissue, all eight CLasMV1 genes were significantly up-regulated (FDR < 0.05) in fruit pith tissue with the value of Log2 fold change ranging from 2.02 to 4.16 (Table 4). 

**Figure 3:**
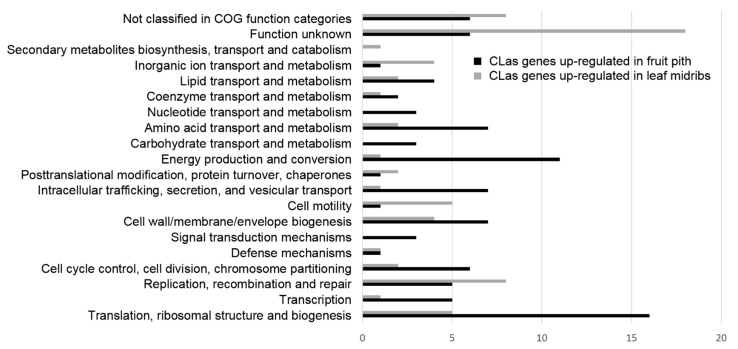
Functional classification of differentially expressed genes (DEGs) of “*Candidatus* Liberibacter asiaticus” in leaf midribs tissue and fruit pith tissue. The DEGs were identified with CLC Genomic Workbench v20 by setting Log2 fold change > |1| and FDR < 0.050. Functional annotation and orthologs assignment of all DEGs were performed with eggNOG-mapper v2 by using Clusters of Orthologous Groups (COG) database.

Differential expression analyses identified 169 differentially expressed genes (DEGs) of CLas between leaf midribs (low-abundant CLasMV1 phage) and fruit piths (high-abundant CLasMV1 phage) (Figure 3 and Table S2). Of 169 CLas DEGs, 101 genes were significantly up-regulated (Log2 fold change > 1 and FDR < 0.05) in fruit pith tissue, while 68 genes were significantly up-regulated in leaf midribs (Figure 3 and Table S2). Functional orthology assignments of 169 DEGs identified a total of nineteen diverse function groups and one group of genes that were not classified in the COG database (Figure 3). A large number of genes involved in cell wall/membrane/envelope biogenesis, translation, ribosomal structure and biogenesis, energy production and conversion, intracellular trafficking, secretion and vesicular transport, amino acid transport and metabolism were up-regulated in fruit pith tissue (Figure 3). In addition to the hypothetical genes with unknown function, genes involved in replication/recombination/repair, cell motility, translation, ribosomal structure and biogenesis, cell wall/membrane/envelope biogenesis, inorganic ion transport and metabolism were mainly up-regulated in leaf midribs tissue (Figure 3).

It was found that four CLas genes involved in Sec pathway, i.e., *SecA* (CD16_00990), *SecB* (CD16_01910), *SecD* (CD16_04035) and *SecY* (CD16_00550), were up-regulated in fruit pith compared to leaf midribs. Noteworthy, a restriction endonuclease subunit S gene (CD16_03585) from Type I restriction–modification system was overexpressed in fruit pith tissue (Supplementary Table S2). Eight genes involved in biogenesis of CLas cell surface structure (peptidoglycan, lipopolysaccharides and pilus) were also up-regulated in fruit pith (Table S2). These included a pilus assembly gene (*TadC*, CD16_02395), a Flp family type IVb pilin gene (CD16_02330), a glycosyl transferase gene (CD16_04000), a peptidoglycan-binding protein gene (CD16_01665), a L,D-transpeptidase family protein (CD16_03055, 23.4-fold), two lipopolysaccharides biosynthesis related genes (CD16_01550 and 05035) and a penicillin-binding protein gene (CD16_02255). In contrast, six genes involved in flagellar biosynthesis (CD16_01255, 02570, 03335, 03350, 03365 and 03420) were down-regulated in fruit pith tissue (Supplementary Table S2).

## 3. Discussion

Although CLas and CLasMV1 phage showed similar distribution in CLas-infected citrus branches, not all CLas cells were infected with CLasMV1 phage. In this study, all psyllids showed CLas-positive after feeding on CLas-infected citrus (also detected as CLasMV1-positive), but only 32.5% (13/40) of the total CLas-infected psyllids showed positive for CLasMV1 (Table 1). This suggests that CLas cells can be acquired by psyllid but that not all CLas cells contained CLasMV1 phage, indicating the uneven infection of CLasMV1 phage in CLas population of citrus leaves. Alternatively, ACP may not be favorable to the multiplication of CLasMV1 in CLas. A previous study found that a small repressor protein produced by the ACP resident endosymbiont *Wolbachia* was able to repress CLas phage (SC1 phage) lytic cycle genes and could play a critical role in the survival of endosymbionts in ACP [19]. Conversely, all dodder tendrils that parasitized on CLas-infected citrus branch (also harbored CLasMV1) were positive for both CLas and CLasMV1 phage (Table 2). This could be due to the larger dose of CLas cells being absorbed by dodder’s haustorium (~400 µm in diameter and >5 infection sites) than the psyllid’s needle-like stylet bundle (~1 µm in diameter and single infection site) [20,21], which can significantly increase the acquisition rate of CLasMV1-infected CLas cells. In addition, a higher density of CLasMV1 phage in the CLas-enriched dodder tendril compared to the parasitizing citrus branch (Table 2) also indicated that the niche of dodder phloem could be beneficial to the multiplication of CLasMV1 in CLas.

The bacteriophage reproduction was involved with two main different life cycles; the lytic cycle, by replicating within the host cell and lysing the host cell to release progeny, or the lysogenic cycle, by the integration of their whole genome into the host genome or the formation of a circular replicon in the host cytoplasm [22]. A previous study found only a partial CLasMV1 genome sequence instead of the whole CLasMV1 phage genome in CLas genomes, indicating the absence of a lysogenic form of CLasMV1 phage [10]. Most members of the *Microviridae* family were not thought to undergo lysogeny, excepting one publication reporting that some microvirus-related proviruses were able to lysogenize their host [23]. In addition, the annotation of CLasMV1 phage genome revealed the absence of integrase-related genes in CLasMV1 genome [10], which also suggested the lack of lysogenic conversion cycle for CLasMV1 phage during the CLasMV1-CLas interaction.

In contrast, CLasMV1 phage may replicate via lytic cycle or as a circular plasmid. Our previous study showed that CLasMV1 phage can replicate up to over 1000 copies per CLas cell in CLas sample [10], indicating a possible high level of lytic cycle activity in CLas strain. Generally, a phage lytic cycle leads to the death of host. However, for the smaller genome size of DNA phages, they had a much more limited coding capacity and needed to rely more heavily upon the host machinery to produce new phage particles [24]. As a result, a *Microvirus* phage, including CLasMV1 phage (a *Microviridae* phage), may not immediately kill their host and may coexist with the host for the majority of the infection stage. This was consistent with our observation in this study wherein CLasMV1 showed a similar distribution pattern to CLas within citrus branches (Figure 2).

CLasMV1 phage could undergo a strong lytic activity in CLas-infected fruit pith tissue. A high-level expression of CLasMV1 genes, especially the MCP gene, was observed in CLas-infected fruit pith tissue (Table 4). The density of the host bacteria population played an important role in determining the optimal infectivity for phage [25]. When the hosts were abundant, the phage performed the delay infection to reduce the competition from others for a nearby resource [25]. This could explain why both CLas and CLasMV1 present at high abundance in fruit pith. With a high abundance of CLas population in fruit pith, CLasMV1 phage may undergo a longer infection before the lysis of the CLas cell.

**Figure 4:**
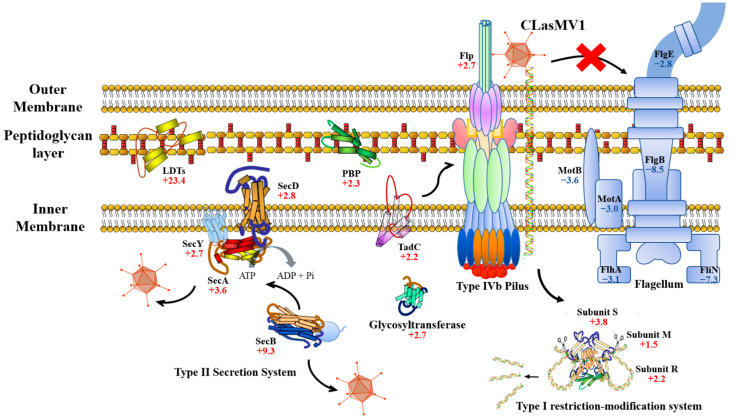
Selective regulation of “*Candidatus* Liberibacter asiaticus” gene response to CLasMV1 phage infection. Values indicate fold change of genes expression during infection. “+”: up-regulation. “−”: down-regulation.

CLasMV1 phage infection significantly induced CLas cell envelope biogenesis gene expression. In particular, an L,D-transpeptidases (LDTs) gene (CD16_03055) of CLas was strongly induced (23.4-fold, FDR = 0.01) in CLasMV1-abundant fruit pith under the stress of highly abundant CLasMV1 phage (Figure 4 and Supplementary Table S2). LDTs were key peptidoglycan-cross-linking enzymes and were found to be involved with bacterial cell wall adaptation to stress, e.g., outer membrane stability and β-lactam antibiotics [26]. Previous studies showed that the CLas-encoded LDTs gene (*ldtP*) was mediated by transcriptional activator LdtR to overcome the osmotic stress in the phloem of the host plant [27,28]. In *Escherichia coli*, the activities of LDTs induced the peptidoglycan remodeling program to increase the overall robustness of the bacterial cell envelope and avoid cell lysis in response to defects in the outer membrane [29]. In addition to LDTs, a glycosyltransferase gene (CD16_04000) involved in the CLas cell wall biogenesis was also overexpressed in CLasMV1-abundant fruit pith (Figure 4, Supplementary Table S2). The glycosyltransferase was known to be involved in the biosynthesis and maintenance of the cell wall [30]. In particular, the glycosyltransferase had been shown to play a critical role in preventing the phage adsorption/infection and protecting *Staphylococcus aureus* against the lytic activity of *Podoviridae* phage [31]. Therefore, in consideration of their important role in cell wall biosynthesis in the stress response, the up-regulation of CLas genes involved in the cell wall or peptidoglycan biogenesis suggested they could play a critical role in the response to CLasMV1 infection by maintaining the CLas cell’s envelope integrity.

CLasMV1 phage infection activated the restriction–modification (RM) system of CLas. In this study, a restriction endonuclease subunit S gene (CD16_03585, 3.8-fold, FDR value = 0.04), belonging to the Type I RM system, was up-regulated in CLasMV1-abundant fruit pith tissue (Figure 4 and Supplementary Table S2). The bacterial RM system was a defense system to prevent foreign DNA infection, particularly against bacteriophage [32]. The Type I RM system complex comprised three subunits, R for restriction, M for modification and S for specificity [33]. The S subunit was necessary for both restriction and modification and was responsible for the recognition of the foreign DNA sequence specific for the RM system [34]. It was also found that a restriction endonuclease subunit M (CD16_01680, 1.5-fold) and a subunit R (CD16_03410, 2.2-fold) were up-regulated in fruit pith tissue, although the fold change was not significant (FDR value > 0.05) (Figure 4 and Supplementary Table S1). The up-regulation of subunit genes involved in the CLas RM system in CLasMV1-abundant fruit pith tissue was believed to protect CLas from CLasMV1 phage infection.

CLasMV1 phage could regulate genes involved in CLas cell surface components to be beneficial for its infection. In this study, CLas genes involved in peptidoglycan-binding, lipopolysaccharides, pilus assembly and Sec pathway systems were up-regulated in fruit pith (Figure 4 and Supplementary Table S2). Large numbers of studies have shown that phage is capable of recognizing the surface components (or receptors) of the assortative host bacteria during initial infection [35,36,37,38,39]. These surface components mainly included lipopolysaccharides, outer membrane porins, flagella proteins and pilus [35,36,37,38,39]. The overexpression of peptidoglycan-binding proteins, lipopolysaccharides and pilus assembly in fruit pith suggest that these CLas cell surface components could be the possible receptor for CLasMV1 phage adsorption (Figure 4). In addition to CLas cell surface components, four Sec-pathway-related genes, including *SecA* (CD16_00990, 3.6-fold), *SecB* (CD16_01910, 9.3-fold), *SecD* (CD16_04035, 2.8-fold) and *SecY* (CD16_00550, 2.7-fold), were up-regulated in fruit pith compared to leaf midribs (Figure 4). The bacterial Sec pathway was an essential and universal export machinery for the translocation of proteins across the cytoplasmic membrane to periplasm and outer membrane [40]. Except for the function of protein export, the components of the Sec pathway were also found to be required for the correct localization of phage proteins and the propagation of bacteriophage [41,42]. In *E. coli*, SecA was required by f1 bacteriophage assembly proteins to reach their proper membrane location [41]. A previous study has shown that a SecB of *E. coli* was necessary for the efficient propagation of phage LL5 [42]. The significant up-regulation of CLas SecB and SecA in CLasMV1-abundant CLas samples suggest they could play an important role in the maintaining and propagation of CLasMV1 phage growth in CLas. In contrast, six genes involved in flagellar biosynthesis (CD16_01255, 02570, 03335, 03350, 03365 and 03420) were down-regulated in fruit pith tissue (Figure 4, Supplementary Table S2). The bacterial flagella were identified as another receptor for specific binding of phage, especially for the flagellotropic bacteriophages belong to the tailed-phage order *Caudovirales* [43]. However, in this study six CLas genes involved in flagella formation of CLas were down-regulated in CLas-abundant fruit pith tissue (Supplementary Table S2), which suggested that the CLas flagella could not be the main receptor for adsorption of CLasMV1 phage on the CLas cell.

## 4. Materials and Methods

### 4.1. Quantification Analyses of CLas and CLasMV1 Phage

The quantification analyses of CLas were performed by SYBR Green Real-time PCR with primers (CLas4G/HLBr) and probes (HLBp) according to a previous study [44]. The quantification analysis of CLasMV1 phage was performed by SYBR Green Real-time PCR with specific primer set CLasMV1-1F/CLasMV1-1R [10]. All PCR assays were performed in CFX Connect Real-time PCR System (Bio-Rad, Hercules, CA, USA). PCR reaction mixture contained 10 μL of iQ™ SYBR^®^ Green Supermix (Bio-Rad), 0.5 μL of each forward and reverse primer (10 μM) and 1 μL of DNA template (~25 ng) in a final volume of 20 μL with the following procedure: 95 °C for 3 min, followed by 40 cycles at 95 °C for 10 s and 60 °C for 30 s. Fluorescence signal was captured at the end of each 60 °C step. The density of CLasMV1 phage was indicated as copy number of CLasMV1 phage per CLas cell by the ΔCt method with CLasMV1-specific primer set CLasMV1-1F/CLasMV1-1R and CLas-specific primer set CLas-4G/HLBr according to a previous study [11], i.e., R = 2^−ΔCt^, ΔCt = Ct (CLasMV1-1F/CLasMV1-1R)−Ct (primer set targeted single copy gene). The Ct value generated by primer set targeted single copy gene was converted from the Ct value generated by primer set CLas-4G/HLBr (targeted three copies of 16S rRNA gene) with the equation: Ct (primer set targeted single copy gene) = Ct (CLas4G/HLBr) + 1.585.

### 4.2. Psyllid and Dodder Acquisition Assay

Both psyllid- and dodder-mediated acquisition assay were performed in this study. For psyllid-mediated acquisition assay, four CLas-infected citrus plants potted for two years, which had been confirmed to harbor CLasMV1 by CLasMV1-specific Real-time PCR (with primer set CLasMV1-1F/CLasMV1-1R from [10], were used as host plants for feeding by psyllids. The colony of CLas-free psyllids were initially derived from adults collected from CLas-free orange jasmine (*Murraya paniculata* (L.) Jack). A total of ten CLas-free adult psyllids were placed in the new flush of each citrus plant with a plant growth chamber and collected for DNA extraction after two-weeks’ feeding.

For dodder-mediated acquisition assay, a total of 20 CLas-infected citrus branches, which were confirmed to be positive for CLasMV1, were collected from different citrus trees located in Huizhou city, Guangdong province, China, and used as host sources for the dodder’s parasitizing. The dodder-mediated acquisition assay was performed according to a previous study [45]. Briefly, all CLas-infected citrus branches were maintained in a 15 mL tube (filled up with the distilled water) and fixed with cotton to keep the leaves in the air. Dodder plants were initially germinated from seeds and grew on CLas-free periwinkle (*Catharanthus roseus* (L.) G. Don). The dodder plants were ready to be used for parasitizing when new tendrils emerged and elongated, and they had over three tendrils (>5 cm each). Each CLas-infected citrus shoot was parasitized by the tendrils from the individual dodder plant. The connection between the dodder tendrils and periwinkle was cut off after the dodder tendril successfully formed the haustoria and parasitized in the citrus branches. Both dodder tendrils and three citrus leaves (closed to the parasitizing site) were sampled simultaneously after 14 days or when the dodder tendrils started to decline. DNA samples extracted from dodder tendrils and citrus leaves samples were used for the quantification analyses of CLas and CLasMV1 phage as described above.

### 4.3. Distribution Analyses of CLasMV1 Phage in CLas-Infected Citrus Branches

To analyze the distribution of CLasMV1 phage, HLB-affected citrus branches of eight citrus cultivars were collected from southern China during January 2020 to January 2021. These included ‘Eureka’ lemon (*Citrus limon* cv. ‘Eureka’), ‘Huangyan’ mandarin (*C. reticulata* Blanco ‘Subcompress’), ‘Jiaokan’ mandarin (‘Tankan’), ‘Shatangju’ mandarin (‘Shatangju’), ‘Wokan’ mandarin (‘Wokan’), ‘Gongkan’ mandarin (‘Gongkan’), ‘Hongjiang’ sweet orange (‘Gailiang Cheng’) and ‘Liu’ sweet orange (C. *sinensis* cv. ‘Liu Cheng’). Citrus branches were collected from citrus trees showing HLB typical symptoms. For each citrus cultivar, 10–12 symptomatic branches were collected from HLB-affected trees. Each branch contained at least one fruit and one new shoot. The symptomatic leaf sample of each HLB-affected branch was initially tested with both CLas-specific Real-time PCR and CLasMV1-specific Real-time PCR. In this study, only the branch that showed Ct < 32 of both CLas and CLasMV1 phage was selected for further analyses. The sampling of different tissue types from individual citrus branch for the distribution analysis of CLas and CLasMV1 phage was referenced from a previous study [45]. A total of six types of tissues from individual citrus branch were collected, including leaves of new flush, mature leaves, leaves adjacent to fruit, peduncle, the central axis of fruit and fruit pith tissue (Supplementary Figure S1). For leaf samples, only the leaf midrib was collected for DNA extraction. The fresh tissue samples of approximately 100 mg were taken from midribs, pedicles, fruit pith and the central axis. All samples were cut into sections of about 1 mm wide and grinded with a MP FastPrep^®^—24 Grinder (MP Biomedicals LLC, Santa Ana, CA, USA). The DNA extraction of all samples was conducted by E. Z. N. A. HP Plant DNA Kit (OMEGA Bio-Tek Co., Guangzhou, China) according to the manufacturer’s manual. The quantification analyses of CLas and CLasMV1 phage were performed as described above. The density of CLas and CLasMV1 phage among different tissues was evaluated by single factor ANOVA (Duncan’s multiple range test) at 95% (*p* < 0.05) confidence interval. All tests were performed using the SPSS Statistic package v19.0 (IBM, Armonk, NY, USA).

### 4.4. Transcriptome Analyses of CLas and CLasMV1 Phage by Dual RNA-Seq

RNA samples were extracted from the leaf midribs (low-abundant CLasMV1 phage) and fruit pith (high-abundant CLasMV1 phage) of CLas-infected citrus branches (three replicates) (Table S1). The quality of total RNA sample was further confirmed by Qubit 2.0 (Thermo Fisher Scientific Inc., Waltham, MA, USA) and Agilent 2100 (Agilent Technologies Inc., Santa Clara, CA, USA). The Dual RNA-seq library preparation was conducted with a TruSeq RNA library Prep Kit (Illumina, San Diego, CA, USA) by removing cytoplasmic and mitochondrial rRNA from total RNA extract. Sequencing was carried out on an Illumina HiSeq 3000 system (Illumina, San Diego, CA, USA) with 150-bp paired-end read. The clean HiSeq reads were aligned separately to the CLas strain A4 genome (CP010804.2) and CLasMV1 genome (CP045566.1) by CLC Genomic workbench v20 (QIAGEN Bioinformatics, Aarhus, Denmark) (length faction = 0.95; similarity fraction = 0.95). Reads mapped to CLasMV1 or CLas were then summarized into count tables of “Total Gene Reads”. The Transcripts Per Kilobase Million (TPM) method was used for the normalization of RNA-seq, i.e., TPM = A × 10^6^ × 1/∑(A), where A = total reads mapped to gene × 10^3^/gene length in bp. Differentially expressed genes (DEGs) between fruit pith HiSeq data and leaf midribs HiSeq data were performed with CLC Genomic Workbench v20. Log2 fold change > |1|and FDR < 0.050 were set as the cut-off values. The functional annotation and orthologs assignment of DEGs were performed with eggNOG-mapper v2 [46].

## 5. Conclusions

In conclusion, this study first observed that CLasMV1 phage and its host bacteria CLas can be acquired by both insect vector ACP and dodder plant. A higher acquisition efficiency of CLasMV1 phage with CLas was observed by dodder than by psyllid. Both CLasMV1 phage and CLas showed similar distribution patterns in the citrus branch by presenting at significantly higher abundance in the fruit pith and/or the center axis tissue. A relatively high expression level of CLasMV1 phage genes, especially the MCP gene, was observed in CLas-abundant fruit pith tissue. CLasMV1 phage infection could cause the disruption of cell wall structure/synthesis and significantly up-regulate the genes involved in the maintaining of CLas cell envelope integrity. The RM system of CLas was also activated to protect CLas from CLasMV1 phage infection. In addition, the regulation of genes in the CLas cell surface structure and Sec pathway by CLasMV1 could also be beneficial for its infection and propagation. The findings of this study provided evidence of a possible lytic induction of CLasMV1 phage in CLas, and gave new insights into the interaction between CLasMV1 phage and CLas.

## Figures and Tables

**Table 1:** Quantification result of “*Candidatus* Liberibacter asiaticus” and CLasMV1 phage in infected citrus hosts and Asian citrus psyllid (ACP).

Plant ID	Ct Value (Plant)		Copy Number of CLasMV1 Phage per CLas Cell	No. of Psyllids			Copy Number of CLasMV1 per CLas Cell *			
	CLas	ClasMV1		Feeding Psyllid	CLas-Positive	CLasMV1-Positive	<1	≈1	>1	Range
CT1	21.55	18.56	23.8	10	10	2	0	1	1	0.98~2.26
CT2	21.37	17.09	58.2	10	10	2	0	2	0	0.98~1.06
CT3	23.74	25.05	1.21	10	10	5	0	1	4	1.04~4.77
CT4	21.58	20.92	4.74	10	10	4	0	0	4	1.77~2.70
Total	-	-	-	40	40	13	0	4	9	-

* Copy number of CLasMV1 phage per CLas cell was calculated with the ΔCt method, i.e., R = 2^−ΔCt^, ΔCt = Ct (ClasMV1-1F/ClasMV1-1R)−Ct (CLas-4G/HLBr)−1.585.

**Table 2:** Quantitative Real-time PCR result of “*Candidatus* Liberibacter asiaticus” and CLasMV1 phage in citrus and its parasitized dodders.

Group	No.	Sample ID	CLas (Ct Value)		CLasMV1 Phage (Ct Value)		Copy Number of CLasMV1 per CLas Cell		
			Citrus	Dodder	Citrus	Dodder	Citrus	Dodder	Ratio (Dodder/Citrus)
CLas-enriched dodder group	1	C912	21.01	19.47	17.88	17.07	26.33	15.83	0.60
	2	C94	21.98	19.04	17.25	13.31	80.01	158.58	1.98
	3	TSZ63	18.35	15.68	15.92	11.55	16.18	52.66	3.26
	4	TSZ51	21.07	17.83	21.75	17.52	1.88	3.73	1.98
	5	TSZ22	20.45	16.97	17.99	13.52	16.58	32.64	1.97
	6	R10	21.06	18.92	26.13	23.28	0.09	0.15	1.64
	(Avg.)	-	20.66	17.99	19.49	16.04	23.51	43.93	1.91
CLas-unenriched dodder group	7	KS21	18.27	20.72	19.16	21.04	1.62	2.40	1.48
	8	KS2	18.45	24.85	17.28	24.93	6.75	2.83	0.42
	9	HZ1	18.15	20.71	24.08	24.56	0.05	0.21	4.26
	10	HZ3	19.89	23.54	16.45	20.47	32.63	25.18	0.77
	11	HZ7	20.25	24.07	17.15	21.95	25.81	13.07	0.51
	12	HZ9	19.41	23.06	16.97	19.86	16.19	27.60	1.71
	13	CH14	19.35	24.45	18.63	24.28	4.94	3.37	0.68
	14	CH2	19.39	22.52	18.95	22.20	4.04	3.77	0.93
	15	TSZ84	20.88	26.43	21.17	25.09	2.45	7.61	3.10
	16	TSZ72	21.13	21.30	20.07	19.65	6.27	9.42	1.50
	17	TSZ74	21.10	25.11	21.34	23.46	2.53	9.44	3.73
	18	TSZ81	22.77	26.19	23.54	25.15	1.76	6.19	3.52
	19	TSZ62	20.77	25.95	21.00	25.10	2.55	5.38	2.11
	20	TSZ23	20.44	23.87	18.59	22.50	10.81	7.77	0.72
	(Avg.)	-	20.02	23.77	19.60	22.87	8.46	8.87	1.82
Total	(Avg.)	-	-	-	-	-	12.97	19.39	-

**Table 3:** General information of leaf midrib and fruit pith samples used for dual RNA-Seq.

Tissue Type	Sample ID	Ct Value *		Copy Number of CLasMV1 Phage Per CLas Cell	Clean Reads *	CLas Reads *	CLasMV1 Reads *
		CLas	CLasMV1				
Fruit pith	F1	18.32	11.50	338.97	126,816,860	272,423	16,474 (6.05%)
	F2	18.86	11.94	363.30	155,302,372	344,849	20,682 (6.00%)
	F3	19.46	12.63	341.32	98,738,248	229,076	13,673 (5.97%)
	(Avg./Total)	18.88 ± 0.33	12.02 ± 0.33	347.86 ± 7.75	380,857,480	846,348	50,829 (6.01%)
Leaf midribs	L1	26.48	26.16	3.75	135,312,504	22,235	125 (0.56%)
	L2	25.86	25.33	4.33	134,505,318	22,368	106 (0.47%)
	L3	26.51	25.94	4.45	138,098,642	22,751	135 (0.59%)
	(Avg./Total)	26.28 ± 0.21	25.81 ± 0.25	4.18 ± 0.22	407,916,464	67,354	366 (0.54%)

* Ct value, cycle threshold value. Clean reads, reads after quality control of raw sequencing data. CLas strain A4 genome (CP010804.2) and CLasMV1 genome (CP045566.1) were used as reference for reads mapping.

**Table 4:** Transcriptional level of ClasMV1 phage genes in leaf midribs and fruit pith.

No.	Gene Locus Tag	Annotation	Gene Length (bp)	TPM *		Log2 Fold Change	FDR *
				Fruit Pith	Leaf midribs		
1	GE519_gp01	Hypothetical protein	486	14,892	1468	3.20	0.000
2	GE519_gp02	Hypothetical protein	282	11,435	849	3.01	0.022
3	GE519_gp03	Hypothetical protein	327	4579	674	2.02	0.049
4	GE519_gp04	Hypothetical protein	483	17,911	2037	3.02	0.013
5	GE519_gp05	Hypothetical protein	2655	719	12	3.74	0.000
6	GE519_gp06	Replication initiation protein	1218	886	18	2.71	0.049
7	GE519_gp07	Hypothetical protein	675	6120	97	4.16	0.000
8	GE519_gp08	Major capsid protein	1428	9880	1145	3.17	0.000
Avg.	-	-	-	8303	787	3.13	

* TPM, Transcripts per kilobase million. FDR, false discovery rate.

## Data Availability

RNA-seq data from CLas-infected leaf midribs and fruit pith samples supporting the findings of this study are available at the National Center for Biotechnology Information under SRA accession PRJNA848066.

## Supplementary Materials

The following supporting information can be downloaded at: https://www.mdpi.com/article/10.3390/ijms231710024/s1.
