# Supplementary material for: Biological Features and In Planta Transcriptomic Analyses of a Microviridae Phage (CLasMV1) in “Candidatus Liberibacter asiaticus”
Source: Int J Mol Sci. 2022 Sep 2;23(17):10024. doi: 10.3390/ijms231710024 (PMC9456138; doi:10.3390/ijms231710024)
Supplement: Supplementary file 1 [file ijms-23-10024-s001.zip › ijms-1884215-supplementary.pdf]

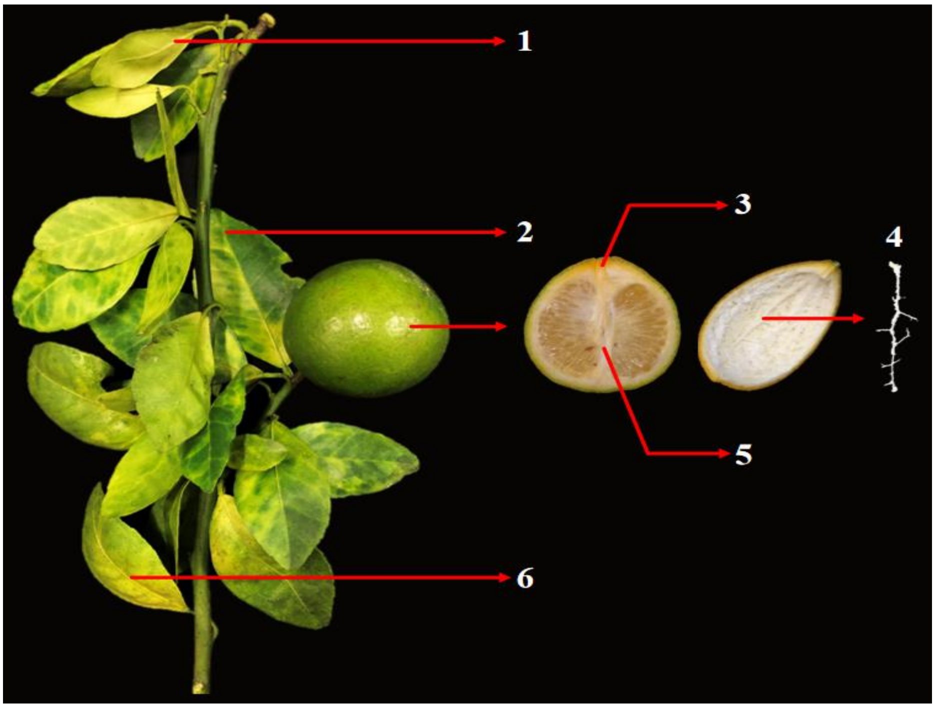

Figure S1. Sampling of different tissues in the branch of HLB-affected *Citrus reticulata* Blanco cv. Gongkan. 1. Leaves from new flush; 2. Leaves adjacent to fruit；3. The peduncle of fruit; 4. Fruit pith; 5. Central axis of fruit； 6. Mature leaves from the bottom of branch.

Table S1. Transcriptional level of "*Candidatus* Liberibacter asiaticus" genes in leaf midribs and fruit pith.

No.	Gene locus tag	locus_tag	Gene length (bp)	TPM*		Log_2 fold change	Fold change	FDR*
				Fruit pith	Leaves mid-ribs			
1	CD16_0000	CD16_00010	360	132	60	1.2	2.3	0.640
2	CD16_00015	CD16_00015	561	103	38	1.5	2.8	0.550
3	CD16_00020	CD16_00020	19	14	0	2.0	4.1	0.860
4	CD16_00025	CD16_00025	1047	43	2247	-2.4	-5.2	0.030
5	CD16_00030	CD16_00030	1227	340	688	-0.9	-1.9	0.080
6	CD16_00035	CD16_00035	378	0	0	NaN	NaN	NaN
7	CD16_00040	CD16_00040	2340	1504	896	0.9	1.8	0.000
8	CD16_00045	CD16_00045	1122	488	277	1.0	1.9	0.100
9	CD16_00050	CD16_00050	774	348	458	-0.3	-1.2	0.760
10	CD16_00055	CD16_00055	1359	1072	629	0.9	1.9	0.009
11	CD16_00060	CD16_00060	627	510	309	0.9	1.8	0.270
12	rpoC	CD16_00065	4197	1449	682	1.2	2.3	0.000
13	rpoB	CD16_00070	4161	2157	1022	1.2	2.3	0.000
14	rplL	CD16_00075	381	3763	2911	0.5	1.4	0.150
15	rplJ	CD16_00080	519	706	843	-0.1	-1.1	0.890
16	rplA	CD16_00085	699	36	62	-0.6	-1.6	0.780
17	rplK	CD16_00090	429	352	101	1.9	3.8	0.200
18	nusG	CD16_00095	534	1068	1529	-0.4	-1.3	0.600
19	secE	CD16_00100	204	285	532	-0.8	-1.8	0.550

20	tuf_1	CD16_00110	1179	404	279	0.7	1.6	0.380
21	mnmA	CD16_00120	1227	402	354	0.3	1.2	0.640
22	CD16_05555	CD16_05555	333	24	391	-3.7	-13.3	0.004
23	CD16_05560	CD16_05560	633	170	0	6.7	101.1	0.510
24	CD16_05565	CD16_05565	249	7	0	1.4	2.6	0.910
25	CD16_00130	CD16_00130	1176	151	185	-0.2	-1.1	0.870
26	CD16_00135	CD16_00135	207	63	0	3.7	13.1	0.720
27	CD16_00140	CD16_00140	771	308	454	-0.4	-1.3	0.590
28	purE	CD16_00145	498	589	877	-0.4	-1.3	0.540
29	CD16_00150	CD16_00150	1065	287	571	-0.9	-1.8	0.320
30	rpmJ	CD16_05570	126	0	0	NaN	NaN	NaN
31	CD16_00155	CD16_00155	291	1913	5344	-1.3	-2.5	0.000
32	CD16_00160	CD16_00160	903	379	529	-0.4	-1.3	0.640
33	CD16_00165	CD16_00165	900	295	581	-0.8	-1.8	0.150
34	clpA	CD16_00170	2397	787	639	0.4	1.3	0.180
35	clpS	CD16_00175	417	821	1767	-1.0	-2.0	0.070
36	CD16_00180	CD16_00180	216	68	0	4.0	16.1	0.710
37	CD16_00185	CD16_00185	786	578	940	-0.6	-1.5	0.210
38	CD16_00190	CD16_00190	237	618	560	0.3	1.2	0.840
39	CD16_00195	CD16_00195	1143	440	554	-0.2	-1.1	0.780
40	CD16_00200	CD16_00200	1029	2069	2840	-0.3	-1.3	0.150
41	CD16_00205	CD16_00205	1197	907	980	0.0	1.0	0.980
42	CD16_00210	CD16_00210	1161	978	434	1.3	2.5	0.002
43	CD16_00215	CD16_00215	774	1367	1442	0.1	1.0	0.940
44	CD16_00225	CD16_00225	309	3225	6027	-0.8	-1.7	0.004
45	CD16_00235	CD16_00235	360	862	1990	-1.1	-2.1	0.020
46	lipB	CD16_00245	774	973	1389	-0.4	-1.3	0.340
47	tgt	CD16_00250	1137	227	323	-0.4	-1.3	0.610
48	CD16_00255	CD16_00255	228	49	0	3.5	11.0	0.740
49	queA	CD16_00260	1083	665	665	0.1	1.1	0.830
50	coaD	CD16_00265	507	451	727	-0.6	-1.5	0.380
51	gyrA	CD16_00270	2733	2356	2515	0.0	1.0	0.870
52	ssb	CD16_00275	480	909	996	0.0	-1.0	0.970
53	uvrA	CD16_00280	2880	167	98	0.9	1.9	0.180
54	glnA	CD16_00285	1377	706	427	0.8	1.8	0.070
55	CD16_00290	CD16_00290	936	511	276	1.0	2.0	0.160
56	rpsD	CD16_00295	621	1743	1771	0.1	1.1	0.940
57	CD16_00300	CD16_00300	1060	106	142	-0.3	-1.2	0.840
58	CD16_05575	CD16_05575	154	0	0	NaN	NaN	NaN
59	fumC	CD16_00305	1392	1316	828	0.8	1.7	0.010
60	alaS	CD16_00310	2697	454	436	0.2	1.1	0.680
61	recA	CD16_00315	1074	468	629	-0.3	-1.2	0.640
62	CD16_00320	CD16_00320	1629	260	293	-0.1	-1.0	0.940
63	rpmE	CD16_00325	225	2044	3477	-0.7	-1.6	0.220

64	CD16_00330	CD16_00330	210	151	107	0.7	1.6	0.810
65	CD16_00345	CD16_00345	1635	748	586	0.5	1.4	0.320
66	secG	CD16_00350	378	568	344	0.8	1.8	0.450
67	CD16_00355	CD16_00355	795	554	985	-0.7	-1.6	0.140
68	CD16_00365	CD16_00365	483	701	807	-0.1	-1.1	0.940
69	CD16_00370	CD16_00370	1032	711	968	-0.3	-1.2	0.720
70	CD16_00375	CD16_00375	1185	236	720	-1.5	-2.7	0.000
71	queE	CD16_00380	636	83	139	-0.6	-1.5	0.710
72	CD16_05580	CD16_05580	161	22	0	2.0	4.1	0.860
73	CD16_00385	CD16_00385	249	213	86	1.3	2.5	0.610
74	CD16_00390	CD16_00390	543	291	198	0.7	1.6	0.590
75	CD16_00395	CD16_00395	933	589	492	0.4	1.3	0.530
76	purD	CD16_00400	1275	460	687	-0.5	-1.4	0.340
77	CD16_00405	CD16_00405	297	528	1471	-1.4	-2.6	0.040
78	CD16_05585	CD16_05585	201	147	0	4.9	29.4	0.640
79	CD16_00410	CD16_00410	474	1449	1621	0.0	-1.0	0.960
80	CD16_00415	CD16_00415	192	1031	1476	-0.4	-1.3	0.640
81	CD16_00420	CD16_00420	816	1627	1311	0.4	1.4	0.230
82	CD16_00425	CD16_00425	789	933	1321	-0.4	-1.3	0.430
83	CD16_00430	CD16_00430	1566	1993	2708	-0.3	-1.2	0.110
84	CD16_00435	CD16_00435	1044	228	587	-1.2	-2.3	0.006
85	CD16_00440	CD16_00440	678	565	1121	-0.9	-1.8	0.030
86	CD16_00450	CD16_00450	294	197	223	-0.1	-1.0	0.970
87	CD16_00455	CD16_00455	294	118	293	-1.2	-2.2	0.440
88	parE	CD16_00460	2061	592	343	0.9	1.9	0.130
89	CD16_00465	CD16_00465	1083	1127	1130	0.1	1.1	0.790
90	CD16_00470	CD16_00470	1149	1024	400	1.5	2.8	0.001
91	CD16_00475	CD16_00475	570	163	308	-0.8	-1.7	0.420
92	ychF	CD16_00480	1104	493	239	1.2	2.2	0.050
93	CD16_00485	CD16_00485	1203	1060	1401	-0.3	-1.2	0.380
94	CD16_00490	CD16_00490	1041	75	63	0.4	1.3	0.840
95	CD16_00495	CD16_00495	5346	506	175	1.7	3.1	0.000
96	CD16_05590	CD16_05590	291	676	370	1.0	2.0	0.380
97	CD16_00500	CD16_00500	297	694	514	0.6	1.5	0.590
98	CD16_00505	CD16_00505	534	911	936	0.1	1.1	0.890
99	typA	CD16_00510	1827	911	475	1.1	2.1	0.001
100	CD16_00515	CD16_00515	1215	1254	807	0.8	1.7	0.020
101	CD16_00520	CD16_00520	225	509	976	-0.8	-1.7	0.470
102	rplQ	CD16_00525	411	1382	639	1.2	2.3	0.040
103	CD16_00530	CD16_00530	1023	1401	1350	0.2	1.1	0.660
104	rpsK	CD16_00535	390	1047	895	0.3	1.3	0.660
105	rpsM	CD16_00540	369	489	647	-0.3	-1.2	0.780
106	CD16_00545	CD16_00545	606	483	326	0.7	1.6	0.380
107	secY	CD16_00550	1335	559	231	1.4	2.7	0.020

108	CD16_00555	CD16_00555	456	24	47	-0.9	-1.9	0.780
109	rpmD	CD16_00560	195	106	111	-0.1	-1.0	0.980
110	rpsE	CD16_00565	600	648	360	1.0	2.0	0.240
111	rplR	CD16_00570	363	459	781	-0.6	-1.5	0.460
112	rplF	CD16_00575	534	655	168	2.1	4.3	0.030
113	rpsH	CD16_00580	390	1827	662	1.6	2.9	0.020
114	rpsN	CD16_00585	306	603	214	1.6	3.1	0.210
115	rplE	CD16_00590	558	1046	193	2.5	5.7	0.002
116	rplX	CD16_00595	309	504	72	2.9	7.3	0.150
117	rplN	CD16_00600	369	228	178	0.5	1.4	0.750
118	rpsQ	CD16_00605	240	497	93	2.5	5.8	0.210
119	rpmC	CD16_00610	204	410	0	6.3	81.0	0.540
120	rplP	CD16_00615	414	1056	733	0.7	1.6	0.340
121	rpsC	CD16_00620	684	1755	736	1.4	2.6	0.003
122	rplV	CD16_00625	396	666	220	1.7	3.3	0.130
123	rpsS	CD16_00630	279	110	0	4.9	29.7	0.640
124	rplB	CD16_00635	837	756	492	0.7	1.7	0.400
125	rplW	CD16_00640	333	968	911	0.2	1.2	0.780
126	rplD	CD16_00645	624	1482	728	1.1	2.2	0.010
127	rplC	CD16_00650	666	121	67	1.0	2.0	0.630
128	rpsJ	CD16_00655	315	573	205	1.6	3.0	0.210
129	tuf_2	CD16_00660	1179	427	297	0.7	1.6	0.340
130	fusA	CD16_00665	2106	1552	2243	-0.4	-1.3	0.090
131	rpsG	CD16_00670	471	59	0	4.7	26.6	0.650
132	CD16_00675	CD16_00675	375	1642	1847	-0.1	-1.1	0.930
133	CD16_00680	CD16_00680	516	1067	299	2.0	3.9	0.010
134	accC	CD16_00685	1332	926	245	2.0	4.1	0.000
135	CD16_00690	CD16_00690	594	610	363	0.8	1.8	0.320
136	CD16_00695	CD16_00695	2463	1480	927	0.8	1.8	0.000
137	clpX	CD16_00700	1275	3093	2408	0.5	1.4	0.020
138	CD16_00705	CD16_00705	651	1764	771	1.3	2.5	0.001
139	ettA	CD16_00710	1620	442	973	-1.0	-2.0	0.040
140	CD16_00715	CD16_00715	954	597	619	0.1	1.1	0.920
141	smpB	CD16_00725	480	447	549	-0.1	-1.1	0.900
142	CD16_00730	CD16_00730	879	1137	863	0.5	1.4	0.290
143	polA	CD16_00735	2931	414	359	0.3	1.3	0.480
144	CD16_00740	CD16_00740	639	187	414	-1.0	-2.0	0.340
145	CD16_00745	CD16_00745	843	483	487	0.1	1.1	0.920
146	CD16_00750	CD16_00750	1047	693	797	-0.1	-1.1	0.920
147	CD16_00755	CD16_00755	606	1130	1904	-0.6	-1.6	0.080
148	CD16_00760	CD16_00760	900	345	242	0.6	1.6	0.460
149	trxB	CD16_00765	966	466	539	-0.1	-1.1	0.910
150	CD16_00770	CD16_00770	1872	1375	1807	-0.3	-1.2	0.230
151	CD16_00775	CD16_00775	1761	386	199	1.1	2.1	0.070

152	CD16_00780	CD16_00780	1233	332	586	-0.7	-1.6	0.120
153	erpA	CD16_00785	330	630	601	0.2	1.1	0.860
154	xth	CD16_00790	846	425	616	-0.4	-1.3	0.510
155	rpoD	CD16_00795	2049	877	558	0.8	1.7	0.020
156	CD16_00800	CD16_00800	999	499	282	0.9	1.9	0.180
157	CD16_00805	CD16_00805	1428	416	184	1.3	2.5	0.030
158	rplM	CD16_00810	468	4163	2983	0.6	1.5	0.030
159	rpsI	CD16_00815	513	4050	1949	1.2	2.3	0.000
160	argC	CD16_00820	936	508	882	-0.7	-1.6	0.220
161	truA	CD16_00825	744	257	118	1.2	2.4	0.340
162	CD16_00830	CD16_00830	912	147	145	0.1	1.1	0.930
163	CD16_00835	CD16_00835	513	369	895	-1.2	-2.2	0.040
164	CD16_00840	CD16_00840	357	329	613	-0.8	-1.7	0.480
165	CD16_00845	CD16_00845	1656	1067	1672	-0.5	-1.4	0.010
166	CD16_00850	CD16_00850	951	731	1218	-0.6	-1.5	0.190
167	CD16_00855	CD16_00855	1296	368	369	0.1	1.1	0.850
168	plsY	CD16_00860	618	238	109	1.3	2.5	0.340
169	dprA	CD16_05595	1166	70	18	2.0	3.9	0.400
170	topA	CD16_00875	2514	464	88	2.5	5.8	0.000
171	CD16_00880	CD16_00880	1008	1266	930	0.6	1.5	0.150
172	CD16_00885	CD16_00885	867	8204	16548	-0.9	-1.9	0.000
173	CD16_00890	CD16_00890	180	240	244	0.2	1.1	0.940
174	CD16_00895	CD16_00895	360	395	1084	-1.3	-2.5	0.070
175	CD16_00905	CD16_00905	534	3590	6004	-0.6	-1.5	0.002
176	CD16_00910	CD16_00910	369	376	303	0.5	1.4	0.730
177	CD16_00920	CD16_00920	684	365	292	0.5	1.4	0.660
178	CD16_00930	CD16_00930	1056	3972	1816	1.3	2.4	0.000
179	CD16_00935	CD16_00935	2418	849	1352	-0.5	-1.5	0.006
180	pheS	CD16_00940	1101	804	1423	-0.7	-1.6	0.070
181	rplT	CD16_00945	372	3393	5273	-0.5	-1.4	0.070
182	rpmI	CD16_00950	204	299	528	-0.7	-1.6	0.610
183	CD16_00955	CD16_00955	567	2802	2636	0.2	1.2	0.620
184	lepA	CD16_00960	1821	2225	4205	-0.8	-1.7	0.000
185	CD16_00965	CD16_00965	822	263	1309	-2.2	-4.4	0.000
186	CD16_00970	CD16_00970	1476	3278	2689	0.4	1.3	0.010
187	CD16_00975	CD16_00975	186	883	704	0.5	1.4	0.690
188	CD16_00980	CD16_00980	426	128	0	5.7	50.5	0.590
189	argJ	CD16_00985	1251	539	621	-0.1	-1.1	0.880
190	secA	CD16_00990	2673	821	252	1.8	3.6	0.000
191	galE	CD16_00995	1002	233	177	0.5	1.5	0.610
192	rpsF	CD16_01000	408	3294	9030	-1.3	-2.5	0.000
193	rpsR	CD16_01005	252	4207	7366	-0.7	-1.6	0.060
194	rplI	CD16_01010	540	2089	1720	0.4	1.3	0.640
195	CD16_01015	CD16_01015	1515	323	561	-0.7	-1.6	0.150

196	alr	CD16_01020	1113	155	136	0.3	1.2	0.830
197	radA	CD16_01025	1440	314	379	-0.2	-1.1	0.800
198	CD16_01030	CD16_01030	471	415	327	0.5	1.4	0.680
199	CD16_01035	CD16_01035	1467	442	519	-0.1	-1.1	0.860
200	CD16_01040	CD16_01040	774	273	168	0.8	1.7	0.480
201	CD16_01045	CD16_01045	546	812	276	1.7	3.1	0.060
202	CD16_01050	CD16_01050	267	98	0	4.7	25.2	0.660
203	CD16_05600	CD16_05600	90	0	0	NaN	NaN	NaN
204	CD16_01055	CD16_01055	1047	357	84	2.2	4.6	0.020
205	CD16_01060	CD16_01060	846	582	465	0.5	1.4	0.480
206	CD16_01065	CD16_01065	930	920	1520	-0.6	-1.5	0.140
207	CD16_01070	CD16_01070	936	579	422	0.6	1.5	0.390
208	queF	CD16_01075	465	268	424	-0.5	-1.4	0.640
209	nusB	CD16_01080	513	548	383	0.7	1.6	0.450
210	CD16_01085	CD16_01085	450	577	534	0.3	1.2	0.760
211	CD16_01090	CD16_01090	615	277	73	2.1	4.2	0.170
212	ribD	CD16_01095	1095	187	344	-0.7	-1.7	0.460
213	CD16_01100	CD16_01100	1302	1624	1443	0.3	1.2	0.360
214	CD16_01105	CD16_01105	1296	1268	958	0.5	1.4	0.120
215	CD16_01110	CD16_01110	516	884	340	1.5	2.8	0.030
216	hemB	CD16_01115	1032	723	591	0.4	1.3	0.450
217	parC	CD16_01120	2262	385	280	0.6	1.5	0.170
218	CD16_01125	CD16_01125	1290	685	300	1.3	2.5	0.010
219	CD16_01130	CD16_01130	363	310	242	0.5	1.4	0.730
220	CD16_01135	CD16_01135	1185	110	73	0.7	1.6	0.620
221	CD16_01140	CD16_01140	450	411	295	0.6	1.5	0.610
222	ribB	CD16_01145	633	742	1144	-0.5	-1.4	0.470
223	CD16_01150	CD16_01150	918	280	456	-0.5	-1.5	0.390
224	CD16_01155	CD16_01155	186	2062	2811	-0.3	-1.3	0.590
225	nhaA	CD16_01160	1182	371	705	-0.8	-1.7	0.150
226	CD16_01165	CD16_01165	795	697	1127	-0.5	-1.5	0.210
227	CD16_01170	CD16_01170	783	724	333	1.2	2.4	0.040
228	CD16_01175	CD16_01175	900	227	242	0.0	1.0	0.970
229	CD16_05605	CD16_05605	525	165	169	0.1	1.1	0.940
230	CD16_01185	CD16_01185	846	438	802	-0.7	-1.7	0.180
231	CD16_01190	CD16_01190	750	1528	2105	-0.3	-1.3	0.330
232	CD16_01195	CD16_01195	957	621	823	-0.3	-1.2	0.620
233	argH	CD16_01200	1422	440	291	0.7	1.7	0.230
234	lysA	CD16_01205	1296	759	358	1.2	2.4	0.007
235	fliP	CD16_01210	741	105	177	-0.6	-1.5	0.640
236	CD16_01215	CD16_01215	519	46	83	-0.7	-1.7	0.730
237	flgH	CD16_01220	717	118	0	6.3	81.1	0.540
238	CD16_01225	CD16_01225	528	65	0	5.1	33.3	0.630
239	flgI	CD16_01230	1110	99	0	6.8	107.6	0.500

240	flgA	CD16_01235	459	107	96	0.3	1.3	0.870
241	flgG	CD16_01240	789	302	581	-0.8	-1.8	0.170
242	CD16_01245	CD16_01245	327	88	198	-1.1	-2.2	0.530
243	flgC	CD16_01250	405	32	111	-1.4	-2.7	0.500
244	flgB	CD16_01255	393	138	1334	-3.1	-8.5	0.000
245	CD16_01260	CD16_01260	1398	2449	2163	0.3	1.2	0.180
246	nth	CD16_01265	684	277	422	-0.5	-1.4	0.600
247	CD16_05610	CD16_05610	420	109	410	-1.8	-3.4	0.210
248	CD16_05615	CD16_05615	165	10	0	1.4	2.6	0.910
249	CD16_01270	CD16_01270	1305	242	167	0.7	1.6	0.400
250	CD16_01275	CD16_01275	1698	513	1132	-1.0	-2.0	0.000
251	CD16_01280	CD16_01280	1323	99	293	-1.4	-2.7	0.150
252	dctA	CD16_01285	1281	1434	1921	-0.3	-1.2	0.240
253	CD16_01290	CD16_01290	1377	323	362	-0.1	-1.0	0.940
254	CD16_01295	CD16_01295	423	1847	1503	0.4	1.3	0.390
255	CD16_01300	CD16_01300	426	81	204	-1.1	-2.2	0.460
256	CD16_01305	CD16_01305	1485	1584	1105	0.7	1.6	0.008
257	CD16_01310	CD16_01310	1086	501	760	-0.5	-1.4	0.480
258	CD16_01315	CD16_01315	1119	262	410	-0.5	-1.4	0.460
259	lptD	CD16_01320	2289	216	199	0.2	1.2	0.710
260	CD16_01325	CD16_01325	954	264	414	-0.5	-1.4	0.460
261	pdxA	CD16_01330	1032	180	317	-0.7	-1.6	0.380
262	rsmA	CD16_01335	855	200	454	-1.1	-2.1	0.150
263	mutL	CD16_01340	1785	518	330	0.8	1.7	0.070
264	CD16_01345	CD16_01345	216	99	104	0.1	1.0	0.980
265	CD16_01350	CD16_01350	1017	207	324	-0.5	-1.4	0.530
266	CD16_01355	CD16_01355	1323	293	283	0.2	1.1	0.850
267	CD16_01360	CD16_01360	243	306	710	-1.1	-2.2	0.330
268	CD16_01365	CD16_01365	861	522	680	-0.3	-1.2	0.620
269	rsmD	CD16_01370	570	370	695	-0.8	-1.7	0.280
270	CD16_01375	CD16_01375	1065	280	405	-0.4	-1.4	0.590
271	CD16_01380	CD16_01380	195	363	782	-1.0	-2.0	0.390
272	prs	CD16_01385	933	396	422	0.1	1.0	0.950
273	CD16_01390	CD16_01390	1089	212	158	0.5	1.4	0.610
274	CD16_01395	CD16_01395	867	128	174	-0.3	-1.3	0.790
275	CD16_01400	CD16_01400	285	662	1291	-0.9	-1.8	0.260
276	CD16_01410	CD16_01410	2463	678	589	0.3	1.3	0.360
277	guaA	CD16_01415	1563	610	348	0.9	1.9	0.050
278	CD16_01420	CD16_01420	1572	373	210	1.0	1.9	0.150
279	CD16_01425	CD16_01425	342	986	1852	-0.8	-1.7	0.070
280	CD16_01430	CD16_01430	567	6623	6106	0.2	1.2	0.200
281	CD16_01435	CD16_01435	576	4791	3563	0.6	1.5	0.020
282	aspS	CD16_01440	1806	928	955	0.1	1.1	0.810
283	carA	CD16_01445	1191	446	514	-0.1	-1.1	0.910

284	CD16_01450	CD16_01450	657	2303	6711	-1.4	-2.7	0.000
285	der	CD16_01455	1413	655	1138	-0.7	-1.6	0.050
286	CD16_01460	CD16_01460	1242	992	908	0.2	1.2	0.590
287	CD16_01465	CD16_01465	435	884	1556	-0.7	-1.6	0.190
288	lnt	CD16_01470	1557	443	294	0.7	1.6	0.250
289	CD16_01475	CD16_01475	963	875	1244	-0.4	-1.3	0.400
290	ybeY	CD16_01480	501	468	521	0.0	-1.0	0.980
291	miaB	CD16_01485	1374	633	756	-0.1	-1.1	0.830
292	tsaB	CD16_01490	621	73	0	5.4	41.9	0.610
293	CD16_01495	CD16_01495	570	1237	499	1.4	2.7	0.010
294	CD16_01500	CD16_01500	537	643	292	1.3	2.4	0.100
295	CD16_01505	CD16_01505	1179	782	903	-0.1	-1.1	0.890
296	argF	CD16_01510	918	458	856	-0.8	-1.7	0.090
297	CD16_01515	CD16_01515	1089	396	479	-0.2	-1.1	0.860
298	CD16_01520	CD16_01520	1011	477	667	-0.4	-1.3	0.580
299	CD16_01525	CD16_01525	897	1364	1795	-0.3	-1.2	0.450
300	rpsU	CD16_01530	231	1013	664	0.7	1.7	0.480
301	CD16_01535	CD16_01535	786	324	359	0.0	-1.0	0.990
302	carB	CD16_01540	3489	639	531	0.4	1.3	0.190
303	greA	CD16_01545	477	2465	1047	1.4	2.6	0.001
304	CD16_01550	CD16_01550	1101	780	316	1.4	2.7	0.005
305	pyk	CD16_01555	1443	475	708	-0.5	-1.4	0.240
306	CD16_01560	CD16_01560	426	129	202	-0.5	-1.4	0.760
307	CD16_01565	CD16_01565	679	1685	2361	-0.4	-1.3	0.250
308	CD16_01570	CD16_01570	2892	1561	1102	0.6	1.6	0.001
309	CD16_01575	CD16_01575	516	156	514	-1.6	-3.0	0.050
310	CD16_01580	CD16_01580	936	459	659	-0.4	-1.3	0.480
311	CD16_01585	CD16_01585	1380	1542	3116	-0.9	-1.9	0.000
312	CD16_01590	CD16_01590	2586	370	479	-0.3	-1.2	0.680
313	acnA	CD16_01595	2691	880	485	1.0	2.0	0.000
314	CD16_01600	CD16_01600	780	4639	9635	-0.9	-1.9	0.000
315	pyrF	CD16_01605	717	535	245	1.3	2.4	0.120
316	dnaN	CD16_01610	1158	671	510	0.5	1.4	0.300
317	pncB	CD16_01615	1257	277	294	0.0	1.0	0.970
318	rpsA	CD16_01625	1731	3890	2568	0.7	1.7	0.000
319	CD16_01630	CD16_01630	654	147	298	-0.9	-1.9	0.410
320	CD16_01635	CD16_01635	1350	271	160	0.9	1.8	0.290
321	CD16_01640	CD16_01640	390	1340	782	0.9	1.9	0.140
322	fabA	CD16_01650	519	895	1232	-0.3	-1.3	0.650
323	fabB	CD16_01655	1221	1522	1294	0.4	1.3	0.280
324	CD16_01660	CD16_01660	804	1275	681	1.0	2.1	0.020
325	CD16_01665	CD16_01665	2022	1502	725	1.2	2.3	0.000
326	CD16_01670	CD16_01670	1275	600	584	0.2	1.1	0.760
327	CD16_01675	CD16_01675	1410	535	263	1.2	2.2	0.060

328	CD16_01680	CD16_01680	1128	496	365	0.6	1.5	0.420
329	CD16_05620	CD16_05620	353	158	61	1.4	2.6	0.580
330	CD16_01685	CD16_01685	261	182	251	-0.3	-1.3	0.860
331	CD16_01690	CD16_01690	2892	380	590	-0.5	-1.4	0.120
332	yacG	CD16_01700	192	2079	2145	0.1	1.1	0.930
333	infA	CD16_01705	333	6301	3265	1.1	2.1	0.000
334	cyoD	CD16_01710	360	373	368	0.2	1.2	0.880
335	cyoC	CD16_01715	633	444	280	0.8	1.7	0.380
336	cyoB	CD16_01720	2016	534	760	-0.4	-1.3	0.280
337	cyoA	CD16_01725	999	530	284	1.0	2.1	0.090
338	rpmG	CD16_01730	168	1150	902	0.4	1.4	0.710
339	CD16_01735	CD16_01735	372	1959	877	1.3	2.4	0.050
340	CD16_01740	CD16_01740	699	356	564	-0.5	-1.4	0.450
341	CD16_01745	CD16_01745	486	306	367	-0.1	-1.1	0.940
342	CD16_01750	CD16_01750	1071	238	204	0.3	1.3	0.710
343	CD16_01755	CD16_01755	1104	944	456	1.2	2.3	0.006
344	rpmH	CD16_05625	135	0	0	NaN	NaN	NaN
345	rnpA	CD16_01760	372	1420	1281	0.3	1.2	0.710
346	yidC	CD16_01765	1746	1402	1055	0.5	1.5	0.030
347	CD16_01770	CD16_01770	639	311	955	-1.5	-2.8	0.001
348	argB	CD16_01775	885	923	734	0.5	1.4	0.430
349	bioB	CD16_01780	987	1552	734	1.2	2.3	0.001
350	CD16_01785	CD16_01785	1146	830	396	1.2	2.2	0.009
351	bioD	CD16_01790	654	690	1664	-1.2	-2.2	0.090
352	CD16_01795	CD16_01795	1272	333	224	0.7	1.6	0.300
353	CD16_01800	CD16_01800	984	417	334	0.5	1.4	0.540
354	CD16_01805	CD16_01805	246	265	91	1.6	3.1	0.490
355	CD16_01810	CD16_01810	306	336	141	1.4	2.6	0.410
356	hslU	CD16_01845	1314	700	934	-0.3	-1.2	0.580
357	hslV	CD16_01850	573	177	651	-1.7	-3.3	0.007
358	CD16_01855	CD16_01855	936	514	258	1.1	2.2	0.100
359	CD16_01860	CD16_01860	1530	509	215	1.4	2.6	0.008
360	CD16_01865	CD16_01865	2379	451	323	0.6	1.5	0.150
361	tsaE	CD16_01870	489	1206	983	0.4	1.3	0.600
362	addB	CD16_01875	3123	270	398	-0.4	-1.3	0.290
363	addA	CD16_01880	3558	252	191	0.5	1.4	0.260
364	trxA	CD16_01885	324	2512	3727	-0.4	-1.3	0.330
365	CD16_01890	CD16_01890	1290	664	830	-0.2	-1.1	0.660
366	CD16_01895	CD16_01895	855	1073	1618	-0.5	-1.4	0.460
367	CD16_01900	CD16_01900	600	497	359	0.6	1.5	0.460
368	dnaQ	CD16_01905	738	644	622	0.2	1.1	0.760
369	secB	CD16_01910	459	826	94	3.2	9.3	0.010
370	CD16_01915	CD16_01915	192	9	0	1.4	2.6	0.910
371	CD16_01920	CD16_01920	699	1103	1245	-0.1	-1.0	0.910

372	gyrB	CD16_01925	2412	1100	1234	0.0	-1.0	0.910
373	CD16_01930	CD16_01930	600	1202	3119	-1.3	-2.4	0.000
374	CD16_01935	CD16_01935	1041	312	188	0.8	1.8	0.290
375	hemJ	CD16_01940	537	181	160	0.3	1.2	0.860
376	CD16_01945	CD16_01945	1272	1139	1335	-0.1	-1.1	0.780
377	mnmE	CD16_01950	1323	798	1396	-0.7	-1.6	0.010
378	mnmG	CD16_01955	1868	845	1039	-0.2	-1.1	0.650
379	rsmG	CD16_01960	663	530	524	0.1	1.1	0.910
380	CD16_01965	CD16_01965	798	414	546	-0.3	-1.2	0.760
381	CD16_01970	CD16_01970	903	502	340	0.7	1.6	0.300
382	CD16_01975	CD16_01975	1038	309	500	-0.6	-1.5	0.500
383	CD16_01980	CD16_01980	498	196	87	1.3	2.4	0.490
384	CD16_01985	CD16_01985	2615	1213	737	0.9	1.8	0.000
385	CD16_01990	CD16_01990	669	548	260	1.2	2.3	0.110
386	CD16_01995	CD16_01995	579	2561	3578	-0.4	-1.3	0.240
387	CD16_02000	CD16_02000	2421	423	171	1.4	2.7	0.004
388	CD16_02005	CD16_02005	615	390	569	-0.4	-1.3	0.610
389	CD16_02010	CD16_02010	696	188	564	-1.4	-2.7	0.020
390	rnhA	CD16_02015	459	145	244	-0.6	-1.5	0.710
391	CD16_02020	CD16_02020	951	189	459	-1.1	-2.2	0.130
392	trpS	CD16_02025	1068	1620	2509	-0.5	-1.4	0.060
393	murJ_1	CD16_02030	1557	311	334	0.0	1.0	0.970
394	CD16_02035	CD16_02035	1257	419	175	1.4	2.6	0.050
395	CD16_02040	CD16_02040	1590	489	690	-0.4	-1.3	0.460
396	CD16_02045	CD16_02045	390	556	555	0.1	1.1	0.920
397	rimP	CD16_02050	579	611	860	-0.4	-1.3	0.650
398	nusA	CD16_02055	1581	644	1172	-0.7	-1.7	0.010
399	infB	CD16_02060	2655	329	172	1.1	2.1	0.020
400	CD16_02065	CD16_02065	387	382	114	1.8	3.6	0.230
401	rpsO	CD16_02070	270	2074	408	2.5	5.6	0.004
402	pnp	CD16_02075	2100	2217	1421	0.8	1.7	0.000
403	CD16_02080	CD16_02080	450	918	584	0.8	1.7	0.330
404	dapE	CD16_02085	1170	571	481	0.4	1.3	0.590
405	dapD	CD16_02090	858	662	405	0.8	1.8	0.160
406	CD16_02095	CD16_02095	468	1038	1578	-0.5	-1.4	0.460
407	rpsB	CD16_02100	837	1219	1273	0.1	1.0	0.940
408	tsf	CD16_02105	891	712	487	0.7	1.6	0.270
409	CD16_02110	CD16_02110	729	104	61	0.9	1.9	0.650
410	CD16_02115	CD16_02115	561	1110	1119	0.1	1.1	0.910
411	uppS	CD16_02120	732	414	479	-0.1	-1.1	0.900
412	CD16_02125	CD16_02125	810	74	53	0.6	1.5	0.760
413	rseP	CD16_02130	1050	1103	946	0.3	1.3	0.700
414	bamA	CD16_02135	2346	560	690	-0.2	-1.1	0.660
415	lpxD	CD16_02140	1044	490	378	0.5	1.4	0.390

416	fabZ	CD16_02145	486	478	181	1.5	2.9	0.150
417	lpxA	CD16_02150	816	67	53	0.4	1.3	0.850
418	CD16_02155	CD16_02155	846	266	204	0.5	1.4	0.610
419	lpxB	CD16_02160	1152	211	117	1.0	2.0	0.410
420	recF	CD16_02165	1128	144	368	-1.3	-2.4	0.030
421	CD16_02170	CD16_02170	807	175	136	0.5	1.4	0.720
422	CD16_02175	CD16_02175	534	223	573	-1.2	-2.4	0.080
423	CD16_02180	CD16_02180	1185	1451	869	0.9	1.8	0.006
424	CD16_02185	CD16_02185	453	244	435	-0.7	-1.6	0.600
425	CD16_02190	CD16_02190	324	774	675	0.3	1.2	0.740
426	recR	CD16_02195	606	400	218	1.0	2.0	0.310
427	murJ_2	CD16_02200	1577	906	1851	-0.9	-1.9	0.001
428	CD16_02205	CD16_02205	1422	513	337	0.7	1.7	0.170
429	CD16_02210	CD16_02210	996	473	371	0.5	1.4	0.590
430	CD16_02215	CD16_02215	489	6095	4953	0.4	1.3	0.090
431	CD16_02225	CD16_02225	651	482	299	0.8	1.8	0.280
432	CD16_02230	CD16_02230	924	408	766	-0.8	-1.7	0.240
433	CD16_02235	CD16_02235	795	252	275	0.0	-1.0	0.980
434	CD16_02240	CD16_02240	969	299	248	0.4	1.3	0.660
435	CD16_02245	CD16_02245	795	565	491	0.3	1.2	0.690
436	CD16_02250	CD16_02250	1104	381	353	0.2	1.2	0.740
437	CD16_02255	CD16_02255	2454	741	391	1.1	2.1	0.001
438	CD16_02260	CD16_02260	2172	842	918	0.0	-1.0	0.980
439	mutS	CD16_02265	2763	345	228	0.7	1.7	0.080
440	CD16_02270	CD16_02270	495	1097	618	1.0	1.9	0.100
441	CD16_02275	CD16_02275	303	1697	2159	-0.2	-1.2	0.760
442	sppA	CD16_02280	882	2750	7182	-1.3	-2.4	0.000
443	CD16_02285	CD16_02285	648	390	884	-1.0	-2.1	0.030
444	CD16_02290	CD16_02290	546	833	765	0.3	1.2	0.730
445	lptB	CD16_02295	789	289	630	-1.0	-2.0	0.070
446	CD16_02300	CD16_02300	786	216	500	-1.1	-2.1	0.120
447	CD16_02305	CD16_02305	1260	595	487	0.4	1.3	0.400
448	CD16_02310	CD16_02310	1104	1035	935	0.3	1.2	0.780
449	CD16_02315	CD16_02315	666	646	550	0.3	1.3	0.650
450	CD16_02320	CD16_02320	195	312	455	-0.3	-1.3	0.840
451	CD16_02325	CD16_02325	579	690	567	0.4	1.3	0.610
452	CD16_02330	CD16_02330	183	19726	7992	1.4	2.7	0.000
453	CD16_02335	CD16_02335	228	4231	3943	0.2	1.2	0.650
454	CD16_02340	CD16_02340	189	864	0	7.3	152.8	0.460
455	CD16_02345	CD16_02345	171	184	0	5.0	31.2	0.640
456	CD16_02350	CD16_02350	177	218	243	0.0	-1.0	1.000
457	CD16_05635	CD16_05635	195	129	111	0.4	1.3	0.900
458	CD16_02355	CD16_02355	363	125	416	-1.6	-3.0	0.150
459	CD16_02360	CD16_02360	531	474	987	-0.9	-1.9	0.110

460	cpaB	CD16_02365	792	365	440	-0.2	-1.1	0.830
461	CD16_02370	CD16_02370	1425	316	619	-0.8	-1.8	0.340
462	CD16_02375	CD16_02375	732	537	326	0.8	1.8	0.340
463	CD16_02380	CD16_02380	1284	804	581	0.6	1.5	0.160
464	CD16_02385	CD16_02385	1452	580	331	0.9	1.9	0.050
465	CD16_02390	CD16_02390	978	582	290	1.1	2.2	0.060
466	CD16_02395	CD16_02395	990	1070	527	1.2	2.2	0.020
467	CD16_02400	CD16_02400	495	14702	19178	-0.3	-1.2	0.040
468	CD16_02405	CD16_02405	783	398	508	-0.2	-1.2	0.830
469	CD16_02410	CD16_02410	723	407	362	0.3	1.2	0.710
470	CD16_02415	CD16_02415	885	360	764	-1.0	-2.0	0.070
471	gndA	CD16_02420	1428	790	444	1.0	1.9	0.030
472	rplS	CD16_02425	426	1776	1070	0.9	1.8	0.100
473	trmD	CD16_02430	711	709	518	0.6	1.5	0.340
474	rimM	CD16_02435	573	883	758	0.4	1.3	0.570
475	rpsP	CD16_02440	351	3760	4120	0.0	1.0	0.990
476	CD16_02445	CD16_02445	1386	760	756	0.1	1.1	0.880
477	CD16_02450	CD16_02450	891	112	149	-0.3	-1.2	0.810
478	ftsY	CD16_02455	966	282	46	2.8	6.7	0.050
479	CD16_02460	CD16_02460	615	425	496	-0.1	-1.1	0.930
480	CD16_02470	CD16_02470	1041	488	547	0.0	-1.0	0.970
481	pstC	CD16_02475	1482	375	832	-1.0	-2.1	0.020
482	pstA	CD16_02480	1278	715	595	0.4	1.3	0.450
483	CD16_02485	CD16_02485	765	906	851	0.2	1.2	0.700
484	phoU	CD16_02490	690	1391	1708	-0.2	-1.1	0.720
485	grpE	CD16_02495	660	2359	2206	0.2	1.2	0.590
486	CD16_02500	CD16_02500	1488	940	320	1.7	3.2	0.001
487	CD16_02505	CD16_02505	1470	875	1101	-0.2	-1.2	0.590
488	CD16_02510	CD16_02510	294	1299	369	1.9	3.7	0.030
489	CD16_02515	CD16_02515	1011	190	823	-2.0	-4.0	0.000
490	mscL	CD16_02520	426	1342	1081	0.5	1.4	0.510
491	gshB	CD16_02525	951	699	458	0.7	1.7	0.230
492	fliF	CD16_02530	1692	383	478	-0.2	-1.1	0.710
493	CD16_02535	CD16_02535	741	495	1119	-1.1	-2.1	0.003
494	CD16_02540	CD16_02540	708	232	463	-0.9	-1.8	0.240
495	CD16_02545	CD16_02545	219	485	999	-0.9	-1.9	0.300
496	flhB	CD16_02550	1065	289	538	-0.7	-1.7	0.300
497	fliG	CD16_02555	1038	204	128	0.8	1.8	0.460
498	fliN	CD16_02560	444	114	0	5.7	50.8	0.590
499	CD16_02565	CD16_02565	957	319	369	-0.1	-1.1	0.910
500	motA	CD16_02570	873	254	818	-1.6	-3.0	0.001
501	flgF	CD16_02575	732	131	235	-0.7	-1.7	0.480
502	fliI	CD16_02580	1317	67	119	-0.7	-1.6	0.590
503	CD16_02585	CD16_02585	1023	189	172	0.3	1.2	0.820

504	surE	CD16_02590	753	1050	229	2.3	4.9	0.002
505	serS	CD16_02595	1293	1350	613	1.3	2.4	0.000
506	CD16_02600	CD16_02600	390	642	1118	-0.7	-1.6	0.230
507	CD16_02605	CD16_02605	327	4141	4318	0.1	1.0	0.910
508	CD16_02610	CD16_02610	459	961	985	0.1	1.1	0.940
509	lipA	CD16_02615	990	668	1126	-0.6	-1.6	0.080
510	lpdA_1	CD16_02620	1446	868	457	1.1	2.1	0.070
511	CD16_02625	CD16_02625	1272	1082	443	1.4	2.6	0.002
512	CD16_02630	CD16_02630	1404	689	447	0.8	1.7	0.100
513	pdhA	CD16_02635	1053	772	1243	-0.6	-1.5	0.150
514	CD16_02640	CD16_02640	318	768	756	0.1	1.1	0.910
515	CD16_02645	CD16_02645	1275	722	272	1.5	2.9	0.002
516	kdsA	CD16_02650	846	261	356	-0.3	-1.3	0.720
517	CD16_02660	CD16_02660	1338	425	827	-0.8	-1.8	0.100
518	purH	CD16_02665	1611	613	515	0.4	1.3	0.530
519	CD16_02670	CD16_02670	624	3178	4305	-0.3	-1.2	0.220
520	CD16_02675	CD16_02675	4731	685	350	1.1	2.1	0.007
521	sbcB	CD16_02680	1416	285	185	0.8	1.7	0.320
522	CD16_02690	CD16_02690	735	264	237	0.3	1.2	0.790
523	CD16_02695	CD16_02695	825	287	425	-0.4	-1.4	0.540
524	CD16_02700	CD16_02700	595	285	362	-0.2	-1.2	0.870
525	tkt	CD16_02705	1986	824	514	0.8	1.7	0.020
526	gap	CD16_02710	1002	1035	345	1.7	3.3	0.000
527	pgk	CD16_02715	1203	236	252	0.0	1.0	0.970
528	CD16_02720	CD16_02720	1020	463	241	1.1	2.2	0.130
529	CD16_02725	CD16_02725	513	1359	930	0.7	1.6	0.290
530	CD16_02735	CD16_02735	651	279	705	-1.2	-2.3	0.080
531	obgE	CD16_02740	1008	210	219	0.1	1.1	0.910
532	rpmA	CD16_02745	273	824	322	1.5	2.8	0.180
533	rplU	CD16_02750	312	2023	2521	-0.2	-1.1	0.720
534	CD16_02760	CD16_02760	276	242	939	-1.8	-3.5	0.060
535	CD16_02765	CD16_02765	870	107	331	-1.5	-2.8	0.130
536	CD16_02775	CD16_02775	201	0	0	NaN	NaN	NaN
537	CD16_02780	CD16_02780	366	22	118	-2.3	-4.8	0.370
538	CD16_02785	CD16_02785	333	18	134	-2.7	-6.4	0.270
539	dnaJ	CD16_02790	1155	1190	775	0.7	1.7	0.060
540	dnaK	CD16_02795	1938	3279	2669	0.4	1.3	0.020
541	CD16_02800	CD16_02800	624	701	418	0.9	1.8	0.230
542	CD16_02810	CD16_02810	1257	149	190	-0.2	-1.2	0.850
543	CD16_02815	CD16_02815	1290	141	219	-0.5	-1.4	0.550
544	CD16_02820	CD16_02820	804	138	412	-1.4	-2.7	0.070
545	CD16_02825	CD16_02825	216	123	0	4.7	26.8	0.650
546	CD16_02830	CD16_02830	189	1059	812	0.5	1.4	0.660
547	CD16_05640	CD16_05640	198	348	435	-0.2	-1.1	0.910

548	CD16_02835	CD16_02835	729	567	1668	-1.4	-2.7	0.010
549	CD16_02840	CD16_02840	675	575	647	0.0	-1.0	0.970
550	CD16_02845	CD16_02845	1188	326	462	-0.4	-1.3	0.610
551	dnaA	CD16_02850	1509	3350	2000	0.9	1.8	0.000
552	rpsT	CD16_02855	273	10878	4262	1.5	2.8	0.000
553	mutM	CD16_02860	870	209	322	-0.5	-1.4	0.610
554	ubiE	CD16_02865	798	218	110	1.1	2.1	0.390
555	ubiB	CD16_02870	1554	288	83	1.9	3.7	0.030
556	coaBC	CD16_02875	1218	548	337	0.8	1.8	0.200
557	CD16_02880	CD16_02880	474	12598	32609	-1.2	-2.4	0.000
558	rirA	CD16_02885	435	384	753	-0.9	-1.8	0.260
559	CD16_02890	CD16_02890	2196	124	100	0.4	1.4	0.640
560	atpH	CD16_02895	561	487	350	0.6	1.5	0.510
561	CD16_02900	CD16_02900	1530	1327	996	0.5	1.5	0.050
562	CD16_02905	CD16_02905	885	759	320	1.4	2.6	0.050
563	atpD	CD16_02910	1437	1063	293	2.0	4.0	0.000
564	atpC	CD16_02915	408	857	539	0.8	1.7	0.280
565	CD16_02925	CD16_02925	1299	523	476	0.3	1.2	0.630
566	CD16_02930	CD16_02930	1512	303	542	-0.7	-1.7	0.380
567	rpoH	CD16_02935	909	1004	1110	0.0	-1.0	0.970
568	CD16_02940	CD16_02940	1023	321	900	-1.3	-2.5	0.002
569	CD16_02945	CD16_02945	906	908	721	0.5	1.4	0.340
570	CD16_02950	CD16_02950	1041	337	717	-1.0	-2.0	0.060
571	CD16_02955	CD16_02955	396	120	389	-1.6	-3.1	0.140
572	CD16_02960	CD16_02960	2235	415	29	3.9	15.1	0.000
573	CD16_02965	CD16_02965	1830	295	373	-0.2	-1.1	0.790
574	hemA	CD16_02970	1206	574	564	0.2	1.1	0.790
575	CD16_02975	CD16_02975	849	215	643	-1.5	-2.8	0.006
576	CD16_02990	CD16_02990	1203	876	450	1.1	2.1	0.020
577	CD16_05645	CD16_05645	843	265	388	-0.4	-1.4	0.570
578	CD16_05650	CD16_05650	996	930	704	0.5	1.5	0.290
579	pgsA	CD16_03000	603	289	717	-1.2	-2.3	0.050
580	uvrC	CD16_03005	1899	401	888	-1.0	-2.0	0.030
581	CD16_03010	CD16_03010	618	23914	21961	0.3	1.2	0.030
582	CD16_03015	CD16_03015	1665	307	483	-0.5	-1.4	0.330
583	CD16_03020	CD16_03020	1317	851	781	0.3	1.2	0.690
584	CD16_03025	CD16_03025	870	242	398	-0.6	-1.5	0.420
585	CD16_03030	CD16_03030	693	367	374	0.1	1.1	0.930
586	CD16_03035	CD16_03035	801	307	164	1.0	2.1	0.300
587	CD16_03040	CD16_03040	816	1020	2066	-0.9	-1.8	0.010
588	CD16_03045	CD16_03045	822	485	790	-0.6	-1.5	0.400
589	CD16_03050	CD16_03050	276	4843	5050	0.1	1.1	0.870
590	CD16_03055	CD16_03055	720	680	31	4.6	23.4	0.010
591	CD16_03060	CD16_03060	954	882	798	0.3	1.2	0.650

592	CD16_03065	CD16_03065	963	191	274	-0.4	-1.3	0.700
593	CD16_03070	CD16_03070	306	788	642	0.4	1.3	0.680
594	CD16_03075	CD16_03075	576	1250	1286	0.1	1.1	0.910
595	rpmB	CD16_03080	294	1086	981	0.3	1.2	0.690
596	gloB	CD16_03085	771	278	313	0.0	-1.0	0.970
597	CD16_03090	CD16_03090	729	416	714	-0.7	-1.6	0.300
598	CD16_03095	CD16_03095	801	812	983	-0.1	-1.1	0.780
599	CD16_03100	CD16_03100	624	844	798	0.2	1.2	0.760
600	CD16_03105	CD16_03105	864	581	455	0.5	1.4	0.390
601	CD16_03110	CD16_03110	699	2548	3974	-0.5	-1.4	0.050
602	CD16_03115	CD16_03115	1794	519	623	-0.1	-1.1	0.790
603	CD16_03120	CD16_03120	951	711	3195	-2.0	-4.1	0.000
604	CD16_03125	CD16_03125	603	378	429	-0.1	-1.1	0.960
605	CD16_03130	CD16_03130	1386	270	32	3.2	9.1	0.010
606	CD16_03135	CD16_03135	579	1219	822	0.7	1.6	0.230
607	CD16_03140	CD16_03140	549	232	160	0.6	1.6	0.640
608	CD16_03145	CD16_03145	618	307	70	2.2	4.7	0.130
609	CD16_03150	CD16_03150	1074	543	528	0.2	1.1	0.870
610	CD16_03155	CD16_03155	213	14	315	-3.8	-14.3	0.060
611	CD16_03160	CD16_03160	741	323	1067	-1.6	-3.0	0.004
612	CD16_03165	CD16_03165	210	2165	1239	0.9	1.9	0.150
613	CD16_03170	CD16_03170	324	1682	2159	-0.2	-1.2	0.680
614	CD16_03175	CD16_03175	588	646	2047	-1.5	-2.9	0.002
615	rsfS	CD16_03180	360	710	184	2.1	4.2	0.070
616	CD16_03185	CD16_03185	1017	262	664	-1.2	-2.3	0.030
617	thiP	CD16_03190	1608	197	311	-0.6	-1.5	0.460
618	thiQ	CD16_03195	705	227	397	-0.7	-1.6	0.480
619	CD16_03200	CD16_03200	1191	248	345	-0.4	-1.3	0.590
620	CD16_03205	CD16_03205	483	143	89	0.7	1.7	0.710
621	CD16_03210	CD16_03210	363	13	62	-1.8	-3.6	0.530
622	ruvC	CD16_03220	510	239	211	0.3	1.2	0.830
623	ruvA	CD16_03225	624	1642	2795	-0.7	-1.6	0.020
624	ruvB	CD16_03230	1005	350	109	1.8	3.5	0.040
625	CD16_03235	CD16_03235	693	261	890	-1.7	-3.1	0.001
626	CD16_03240	CD16_03240	303	410	786	-0.8	-1.8	0.420
627	CD16_03245	CD16_03245	816	969	1586	-0.6	-1.5	0.150
628	tolB	CD16_03250	1344	1049	1724	-0.6	-1.5	0.030
629	CD16_03255	CD16_03255	483	732	728	0.1	1.1	0.910
630	tilS	CD16_03260	1272	333	464	-0.3	-1.3	0.620
631	hflB	CD16_03265	1980	681	295	1.3	2.5	0.070
632	CD16_03270	CD16_03270	1347	857	926	0.0	1.0	0.960
633	CD16_03275	CD16_03275	396	679	276	1.4	2.7	0.140
634	CD16_03280	CD16_03280	633	532	275	1.1	2.1	0.150
635	CD16_03285	CD16_03285	834	387	1017	-1.3	-2.4	0.007

636	CD16_03290	CD16_03290	822	590	714	-0.2	-1.1	0.780
637	CD16_03295	CD16_03295	843	931	1477	-0.5	-1.4	0.150
638	CD16_03300	CD16_03300	885	1154	3365	-1.4	-2.7	0.000
639	fabD	CD16_03305	945	1313	1263	0.2	1.1	0.690
640	fabG	CD16_03310	744	1249	588	1.2	2.3	0.010
641	CD16_03315	CD16_03315	258	2539	2364	0.2	1.2	0.730
642	fabF	CD16_03320	1272	2212	1775	0.5	1.4	0.110
643	mltG	CD16_03325	999	756	568	0.5	1.4	0.300
644	CD16_03335	CD16_03335	1359	604	4859	-2.9	-7.3	0.000
645	CD16_03340	CD16_03340	651	166	430	-1.3	-2.4	0.160
646	CD16_03345	CD16_03345	1032	149	256	-0.6	-1.5	0.540
647	CD16_03350	CD16_03350	1191	68	277	-1.8	-3.6	0.010
648	CD16_03355	CD16_03355	1194	122	74	0.9	1.8	0.530
649	CD16_03360	CD16_03360	672	559	592	0.1	1.0	0.950
650	CD16_03365	CD16_03365	1266	188	575	-1.5	-2.8	0.020
651	flgK	CD16_03370	1443	148	91	0.8	1.8	0.460
652	CD16_03375	CD16_03375	1074	511	390	0.5	1.4	0.460
653	flaF	CD16_03380	345	682	824	-0.1	-1.1	0.910
654	CD16_03385	CD16_03385	462	183	140	0.5	1.4	0.790
655	flgD	CD16_03390	396	190	0	6.2	72.4	0.550
656	fliQ	CD16_03395	267	49	0	3.7	13.2	0.720
657	CD16_05655	CD16_05655	108	0	0	NaN	NaN	NaN
658	CD16_05660	CD16_05660	330	71	0	4.5	22.2	0.670
659	CD16_05665	CD16_05665	210	16	0	2.0	4.1	0.860
660	dcm	CD16_03405	252	137	349	-1.2	-2.3	0.360
661	CD16_03410	CD16_03410	333	262	130	1.1	2.2	0.550
662	CD16_03415	CD16_03415	201	44	0	3.3	10.1	0.760
663	flhA	CD16_03420	2079	76	253	-1.6	-3.1	0.009
664	fliR	CD16_03425	747	234	558	-1.1	-2.2	0.100
665	CD16_03430	CD16_03430	414	50	54	0.0	-1.0	1.000
666	CD16_03435	CD16_03435	339	43	0	4.0	15.6	0.710
667	CD16_03440	CD16_03440	372	56	472	-2.9	-7.6	0.010
668	CD16_03445	CD16_03445	531	47	162	-1.7	-3.2	0.210
669	folD	CD16_03450	921	1496	2331	-0.5	-1.4	0.090
670	pgl	CD16_03455	705	359	281	0.5	1.4	0.650
671	zwf	CD16_03460	1479	295	120	1.4	2.7	0.080
672	CD16_03465	CD16_03465	1386	526	806	-0.5	-1.4	0.460
673	CD16_03470	CD16_03470	2865	1447	469	1.7	3.3	0.000
674	bamE	CD16_03475	480	2853	3035	0.0	1.0	0.950
675	CD16_03480	CD16_03480	504	225	470	-0.9	-1.9	0.550
676	plsX	CD16_03485	1095	918	240	2.1	4.2	0.000
677	fabH	CD16_03490	975	1124	785	0.6	1.6	0.150
678	CD16_03495	CD16_03495	345	551	252	1.2	2.3	0.320
679	CD16_03500	CD16_03500	618	5352	10263	-0.8	-1.8	0.000

680	CD16_03505	CD16_03505	945	327	448	-0.3	-1.2	0.800
681	CD16_03515	CD16_03515	972	565	319	1.0	2.0	0.090
682	CD16_03550	CD16_03550	1026	287	408	-0.4	-1.3	0.600
683	trkD	CD16_03555	1887	655	518	0.5	1.4	0.280
684	CD16_03560	CD16_03560	1116	228	0	7.9	240.9	0.410
685	dnaE	CD16_03565	3675	262	196	0.5	1.5	0.350
686	CD16_03570	CD16_03570	1947	323	346	0.0	1.0	0.980
687	CD16_03575	CD16_03575	972	824	542	0.7	1.7	0.120
688	CD16_03580	CD16_03580	2025	1063	1433	-0.3	-1.2	0.260
689	CD16_03585	CD16_03585	1281	304	87	1.9	3.8	0.040
690	CD16_05670	CD16_05670	177	9	0	1.4	2.6	0.910
691	CD16_03600	CD16_03600	741	3109	2352	0.5	1.5	0.030
692	CD16_03605	CD16_03605	1155	703	811	-0.1	-1.1	0.890
693	gluP	CD16_03610	1263	352	378	0.0	1.0	0.980
694	CD16_03620	CD16_03620	342	2047	3807	-0.8	-1.7	0.050
695	CD16_03625	CD16_03625	1059	441	810	-0.8	-1.7	0.180
696	CD16_03630	CD16_03630	975	313	291	0.2	1.2	0.800
697	CD16_03635	CD16_03635	849	454	515	-0.1	-1.0	0.950
698	CD16_03640	CD16_03640	336	3257	4306	-0.3	-1.2	0.530
699	groL	CD16_03645	1656	5512	5960	0.0	1.0	0.950
700	CD16_03650	CD16_03650	195	17	0	2.0	4.1	0.860
701	CD16_03665	CD16_03665	366	1456	947	0.8	1.7	0.220
702	CD16_03670	CD16_03670	558	1776	352	2.5	5.5	0.000
703	CD16_03675	CD16_03675	597	393	325	0.4	1.3	0.720
704	CD16_03680	CD16_03680	1191	392	203	1.1	2.1	0.090
705	nuoE	CD16_03685	657	441	893	-0.9	-1.9	0.120
706	nuoF	CD16_03690	1278	64	85	-0.3	-1.2	0.840
707	CD16_03695	CD16_03695	2103	443	197	1.3	2.5	0.006
708	nuoH	CD16_03700	1047	396	330	0.4	1.3	0.650
709	nuoI	CD16_03705	492	784	482	0.8	1.8	0.340
710	CD16_03710	CD16_03710	600	472	1087	-1.1	-2.1	0.050
711	nuoK	CD16_03715	309	167	70	1.4	2.6	0.590
712	nuoL	CD16_03720	2001	223	283	-0.2	-1.2	0.740
713	CD16_03725	CD16_03725	1500	418	380	0.3	1.2	0.690
714	nuoN	CD16_03730	1437	610	317	1.1	2.1	0.020
715	CD16_03735	CD16_03735	759	1028	745	0.6	1.5	0.200
716	CD16_03740	CD16_03740	1680	871	662	0.5	1.4	0.130
717	CD16_03745	CD16_03745	1350	785	466	0.9	1.8	0.040
718	CD16_03750	CD16_03750	1254	447	276	0.8	1.8	0.160
719	CD16_03755	CD16_03755	687	304	321	0.0	1.0	0.970
720	ubiG	CD16_03760	765	641	427	0.7	1.6	0.350
721	CD16_03765	CD16_03765	1236	378	567	-0.5	-1.4	0.410
722	prfA	CD16_03770	1074	993	2086	-1.0	-2.0	0.000
723	prmC	CD16_03775	882	826	1431	-0.7	-1.6	0.080

724	CD16_03780	CD16_03780	633	1956	1642	0.4	1.3	0.390
725	clpB	CD16_03785	2562	1111	892	0.4	1.4	0.080
726	CD16_03790	CD16_03790	1959	591	524	0.3	1.2	0.630
727	CD16_03825	CD16_03825	306	355	141	1.4	2.6	0.390
728	CD16_03830	CD16_03830	985	290	109	1.5	2.8	0.120
729	yidD	CD16_03835	420	495	636	-0.2	-1.2	0.850
730	folE	CD16_03840	588	985	2034	-0.9	-1.9	0.008
731	CD16_03845	CD16_03845	624	4344	1081	2.1	4.4	0.000
732	CD16_03850	CD16_03850	261	256	248	0.1	1.1	0.950
733	CD16_03855	CD16_03855	195	257	0	5.6	47.7	0.590
734	guaB	CD16_03860	1482	1092	2055	-0.8	-1.7	0.000
735	CD16_05675	CD16_05675	315	422	563	-0.3	-1.2	0.820
736	CD16_03865	CD16_03865	648	377	1174	-1.5	-2.9	0.003
737	CD16_03870	CD16_03870	402	1169	1455	-0.2	-1.2	0.710
738	CD16_03875	CD16_03875	711	5159	5882	-0.1	-1.1	0.730
739	CD16_03880	CD16_03880	312	1733	1283	0.6	1.5	0.450
740	CD16_03885	CD16_03885	276	2179	313	2.9	7.4	0.001
741	CD16_03890	CD16_03890	1056	737	456	0.8	1.8	0.080
742	CD16_03895	CD16_03895	1422	1553	1130	0.6	1.5	0.070
743	CD16_03900	CD16_03900	624	1119	1512	-0.3	-1.2	0.500
744	radC	CD16_03905	698	998	1270	-0.2	-1.2	0.710
745	map	CD16_03910	822	1672	646	1.5	2.9	0.020
746	CD16_03915	CD16_03915	1317	584	897	-0.5	-1.4	0.280
747	recJ	CD16_03920	1803	198	340	-0.7	-1.6	0.290
748	CD16_03925	CD16_03925	1239	1613	2534	-0.5	-1.4	0.009
749	CD16_03930	CD16_03930	2301	227	112	1.1	2.2	0.110
750	CD16_03935	CD16_03935	807	374	53	2.9	7.3	0.030
751	CD16_03940	CD16_03940	816	209	219	0.1	1.1	0.960
752	CD16_03945	CD16_03945	291	59	0	4.1	17.3	0.700
753	CD16_03950	CD16_03950	267	5	84	-3.0	-7.8	0.290
754	CD16_03955	CD16_03955	480	85	179	-1.0	-2.0	0.550
755	CD16_03960	CD16_03960	669	564	359	0.8	1.7	0.390
756	purU	CD16_03965	867	442	502	-0.1	-1.1	0.940
757	CD16_03970	CD16_03970	207	109	212	-0.8	-1.8	0.690
758	CD16_03975	CD16_03975	237	5123	2109	1.4	2.6	0.002
759	CD16_03980	CD16_03980	1227	89	18	2.4	5.2	0.240
760	CD16_03985	CD16_03985	1881	1166	371	1.8	3.4	0.000
761	CD16_03990	CD16_03990	1449	435	329	0.5	1.4	0.380
762	CD16_03995	CD16_03995	1791	621	913	-0.4	-1.3	0.150
763	CD16_04000	CD16_04000	1872	872	350	1.5	2.7	0.000
764	rfbC	CD16_04005	597	1413	1355	0.2	1.2	0.700
765	rfbB	CD16_04010	1077	314	223	0.6	1.6	0.390
766	rfbD	CD16_04015	873	363	175	1.2	2.3	0.180
767	rfbA	CD16_04020	879	202	148	0.6	1.5	0.630

768	CD16_04025	CD16_04025	1209	304	232	0.5	1.4	0.590
769	CD16_04030	CD16_04030	1851	843	471	1.0	2.0	0.002
770	secD	CD16_04035	2502	654	254	1.5	2.8	0.000
771	murA	CD16_04045	1293	660	1298	-0.9	-1.8	0.010
772	CD16_04050	CD16_04050	1491	809	1200	-0.5	-1.4	0.150
773	CD16_04055	CD16_04055	1227	125	36	1.9	3.7	0.210
774	CD16_04060	CD16_04060	1359	131	359	-1.3	-2.5	0.090
775	CD16_05680	CD16_05680	171	0	0	NaN	NaN	NaN
776	CD16_04070	CD16_04070	711	1667	2793	-0.6	-1.5	0.020
777	CD16_04075	CD16_04075	2844	1442	1203	0.4	1.3	0.030
778	CD16_04080	CD16_04080	1125	277	134	1.1	2.2	0.170
779	CD16_04085	CD16_04085	831	1125	1608	-0.4	-1.3	0.610
780	CD16_04090	CD16_04090	549	2754	2465	0.3	1.2	0.400
781	CD16_04095	CD16_04095	369	3219	3555	0.0	-1.0	0.960
782	CD16_04105	CD16_04105	399	1396	1159	0.4	1.3	0.590
783	lepB	CD16_04110	747	1248	1290	0.1	1.1	0.870
784	CD16_04115	CD16_04115	684	749	1014	-0.3	-1.3	0.710
785	CD16_04120	CD16_04120	936	408	485	-0.1	-1.1	0.850
786	glmU	CD16_04125	1329	701	740	0.1	1.0	0.930
787	glmS	CD16_04130	1827	396	227	0.9	1.9	0.110
788	CD16_04135	CD16_04135	687	269	539	-0.9	-1.8	0.240
789	recG	CD16_04140	2103	179	310	-0.7	-1.6	0.270
790	CD16_04145	CD16_04145	297	354	656	-0.8	-1.7	0.420
791	mfd	CD16_04150	3564	259	213	0.4	1.3	0.420
792	recO	CD16_04155	723	408	269	0.7	1.6	0.420
793	CD16_04160	CD16_04160	1257	699	499	0.6	1.5	0.180
794	CD16_04165	CD16_04165	744	164	59	1.6	3.0	0.300
795	CD16_04170	CD16_04170	2058	439	264	0.8	1.8	0.080
796	CD16_04175	CD16_04175	573	349	341	0.2	1.1	0.920
797	CD16_04180	CD16_04180	1179	302	518	-0.7	-1.6	0.230
798	CD16_04185	CD16_04185	615	1186	2621	-1.0	-2.0	0.004
799	CD16_04190	CD16_04190	795	989	820	0.4	1.3	0.490
800	CD16_04195	CD16_04195	531	533	328	0.8	1.8	0.350
801	hflK	CD16_04200	1071	949	615	0.8	1.7	0.080
802	CD16_04205	CD16_04205	909	439	476	0.0	-1.0	0.970
803	serB	CD16_04210	894	258	612	-1.1	-2.1	0.030
804	miaA	CD16_04215	912	307	361	-0.1	-1.1	0.890
805	CD16_04220	CD16_04220	603	1562	771	1.2	2.2	0.020
806	CD16_04225	CD16_04225	648	2206	1547	0.6	1.6	0.070
807	CD16_04230	CD16_04230	1059	425	564	-0.3	-1.2	0.710
808	CD16_04235	CD16_04235	690	352	793	-1.1	-2.1	0.020
809	CD16_04240	CD16_04240	1197	886	310	1.6	3.1	0.000
810	grxD	CD16_04245	321	531	67	3.0	8.2	0.120
811	CD16_04250	CD16_04250	240	453	276	0.8	1.8	0.590

812	purL	CD16_04255	2214	560	700	-0.2	-1.2	0.550
813	purQ	CD16_04260	660	830	930	0.0	-1.0	0.970
814	purS	CD16_04265	255	535	517	0.2	1.1	0.910
815	CD16_04270	CD16_04270	768	945	1103	-0.1	-1.1	0.840
816	CD16_04275	CD16_04275	1308	984	1079	0.0	-1.0	0.960
817	rpe	CD16_04280	675	444	748	-0.6	-1.6	0.370
818	CD16_05690	CD16_05690	171	23	0	2.4	5.4	0.830
819	ruvX	CD16_04285	483	127	232	-0.7	-1.6	0.630
820	gatC	CD16_04290	288	674	906	-0.3	-1.2	0.740
821	gatA	CD16_04295	1482	1146	648	0.9	1.9	0.003
822	gatB	CD16_04300	1503	653	378	0.9	1.9	0.030
823	CD16_04305	CD16_04305	366	153	421	-1.3	-2.5	0.240
824	CD16_05695	CD16_05695	168	47	0	3.0	8.0	0.780
825	CD16_04310	CD16_04310	369	5635	6210	0.0	-1.0	0.970
826	CD16_04315	CD16_04315	609	2742	18587	-2.6	-6.2	0.000
827	CD16_04320	CD16_04320	378	914	1328	-0.4	-1.3	0.590
828	CD16_04325	CD16_04325	285	17	0	2.5	5.7	0.830
829	CD16_05700	CD16_05700	744	144	58	1.4	2.6	0.380
830	CD16_04340	CD16_04340	219	914	1377	-0.5	-1.4	0.630
831	CD16_04345	CD16_04345	414	13	0	2.7	6.5	0.810
832	CD16_04355	CD16_04355	456	462	773	-0.6	-1.5	0.460
833	CD16_04360	CD16_04360	558	346	1086	-1.5	-2.9	0.004
834	CD16_04365	CD16_04365	201	23	0	2.5	5.6	0.830
835	uvrB	CD16_04370	2418	158	218	-0.3	-1.2	0.620
836	CD16_04375	CD16_04375	459	151	94	0.8	1.7	0.690
837	CD16_04380	CD16_04380	783	2027	1113	1.0	2.0	0.002
838	CD16_04390	CD16_04390	378	241	342	-0.4	-1.3	0.720
839	CD16_04395	CD16_04395	330	505	917	-0.8	-1.7	0.380
840	CD16_04400	CD16_04400	237	47	0	3.6	12.1	0.730
841	CD16_04405	CD16_04405	441	507	346	0.7	1.6	0.550
842	CD16_04410	CD16_04410	1689	166	220	-0.3	-1.2	0.720
843	CD16_05705	CD16_05705	186	21	0	2.1	4.2	0.860
844	trmA	CD16_04415	1116	224	292	-0.3	-1.2	0.720
845	CD16_04420	CD16_04420	282	44	614	-3.7	-12.7	0.002
846	CD16_04425	CD16_04425	327	244	861	-1.7	-3.3	0.008
847	CD16_04430	CD16_04430	915	1665	5106	-1.5	-2.8	0.000
848	thrS	CD16_04435	1959	933	624	0.7	1.6	0.030
849	CD16_04440	CD16_04440	213	160	404	-1.3	-2.4	0.420
850	CD16_04445	CD16_04445	378	0	0	NaN	NaN	NaN
851	CD16_04450	CD16_04450	1227	122	18	2.8	7.1	0.160
852	CD16_04460	CD16_04460	300	587	658	0.0	-1.0	1.000
853	CD16_04465	CD16_04465	2253	283	223	0.5	1.4	0.530
854	CD16_04470	CD16_04470	588	925	1038	0.0	-1.0	0.960
855	CD16_04475	CD16_04475	687	1106	191	2.7	6.3	0.001

856	pyrC	CD16_04480	1050	464	514	0.0	-1.0	0.970
857	CD16_04485	CD16_04485	909	1311	1919	-0.4	-1.3	0.170
858	CD16_04490	CD16_04490	351	2911	4545	-0.5	-1.4	0.080
859	CD16_04495	CD16_04495	348	336	438	-0.3	-1.2	0.810
860	mvaD	CD16_04500	1068	137	166	-0.1	-1.1	0.920
861	CD16_04505	CD16_04505	975	248	582	-1.1	-2.2	0.010
862	CD16_04510	CD16_04510	1014	61	0	5.9	61.2	0.570
863	CD16_04515	CD16_04515	1198	178	36	2.4	5.1	0.110
864	CD16_04520	CD16_04520	1014	197	216	0.0	1.0	0.970
865	CD16_04525	CD16_04525	1050	274	84	1.8	3.6	0.070
866	CD16_04530	CD16_04530	492	161	219	-0.3	-1.3	0.840
867	CD16_04535	CD16_04535	1665	320	224	0.6	1.6	0.260
868	CD16_04540	CD16_04540	465	1830	1736	0.2	1.2	0.680
869	CD16_05710	CD16_05710	192	26	0	2.5	5.6	0.830
870	CD16_05715	CD16_05715	1257	198	207	0.1	1.0	0.960
871	CD16_04560	CD16_04560	5487	1305	436	1.7	3.2	0.000
872	queC	CD16_04565	723	303	301	0.1	1.1	0.930
873	queD	CD16_04570	357	493	490	0.1	1.1	0.910
874	CD16_04575	CD16_04575	1479	309	238	0.5	1.4	0.460
875	CD16_04580	CD16_04580	1044	1060	294	2.0	3.9	0.000
876	CD16_04585	CD16_04585	699	346	247	0.6	1.5	0.540
877	CD16_04590	CD16_04590	198	209	335	-0.6	-1.5	0.720
878	hemC	CD16_04595	924	363	280	0.5	1.4	0.580
879	tsaD	CD16_04600	1092	298	402	-0.3	-1.2	0.720
880	CD16_04605	CD16_04605	990	141	87	0.8	1.8	0.530
881	CD16_04610	CD16_04610	432	385	349	0.3	1.2	0.860
882	sdhC	CD16_04615	390	761	110	2.9	7.3	0.030
883	sdhD	CD16_04620	393	632	495	0.5	1.4	0.620
884	CD16_04625	CD16_04625	1836	1108	760	0.7	1.6	0.010
885	sdhB	CD16_04630	780	591	0	8.8	440.7	0.350
886	CD16_04635	CD16_04635	489	600	676	-0.1	-1.0	0.960
887	zapE	CD16_04640	1215	653	1144	-0.7	-1.6	0.120
888	mdh	CD16_04645	963	2075	2496	-0.1	-1.1	0.660
889	sucC	CD16_04650	1197	963	1232	-0.2	-1.2	0.590
890	sucD	CD16_04655	903	893	294	1.8	3.4	0.001
891	CD16_04660	CD16_04660	2874	1358	872	0.8	1.7	0.000
892	odhB	CD16_04665	1311	732	495	0.7	1.6	0.270
893	lpdA_2	CD16_04670	1401	404	405	0.1	1.1	0.840
894	rpiA	CD16_04675	696	374	157	1.4	2.6	0.160
895	gorA	CD16_04680	1386	401	377	0.2	1.2	0.780
896	CD16_04685	CD16_04685	516	2685	5101	-0.8	-1.7	0.001
897	CD16_04690	CD16_04690	448	3110	3866	-0.2	-1.1	0.660
898	sufB	CD16_04695	1470	1355	2531	-0.8	-1.7	0.000
899	sufC	CD16_04700	750	1103	1866	-0.6	-1.6	0.110

900	sufD	CD16_04705	1287	831	787	0.2	1.2	0.760
901	sufS	CD16_04710	1221	739	1490	-0.9	-1.8	0.001
902	CD16_04715	CD16_04715	357	500	60	3.1	8.5	0.120
903	sufA	CD16_04720	351	317	246	0.5	1.4	0.700
904	CD16_04725	CD16_04725	1059	268	329	-0.2	-1.1	0.830
905	genX	CD16_04730	1068	420	244	0.9	1.9	0.160
906	efp	CD16_04735	570	599	348	0.9	1.9	0.200
907	CD16_04740	CD16_04740	297	270	1038	-1.8	-3.4	0.005
908	CD16_04745	CD16_04745	477	2597	4025	-0.5	-1.4	0.080
909	CD16_05720	CD16_05720	339	223	457	-0.8	-1.8	0.480
910	CD16_05725	CD16_05725	72	0	0	NaN	NaN	NaN
911	CD16_04755	CD16_04755	573	1103	2090	-0.8	-1.8	0.010
912	hemF	CD16_04765	924	1446	1437	0.1	1.1	0.720
913	CD16_04770	CD16_04770	684	434	288	0.7	1.7	0.400
914	CD16_04775	CD16_04775	801	753	601	0.5	1.4	0.560
915	CD16_04780	CD16_04780	630	355	208	0.9	1.9	0.370
916	CD16_04785	CD16_04785	336	110	193	-0.6	-1.6	0.710
917	CD16_04790	CD16_04790	729	1215	1135	0.2	1.2	0.700
918	CD16_04795	CD16_04795	801	273	162	0.9	1.8	0.460
919	CD16_04800	CD16_04800	258	2093	2132	0.1	1.1	0.910
920	CD16_05730	CD16_05730	294	13275	1471	3.3	9.8	0.000
921	CD16_04805	CD16_04805	282	11435	849	3.9	14.5	0.000
922	CD16_04810	CD16_04810	327	4579	674	2.9	7.5	0.000
923	CD16_04815	CD16_04815	483	17911	2037	3.3	9.6	0.000
924	CD16_04820	CD16_04820	180	3099	963	1.8	3.5	0.010
925	CD16_04825	CD16_04825	522	1154	538	1.2	2.3	0.070
926	CD16_04830	CD16_04830	531	909	984	0.0	1.0	0.980
927	CD16_04835	CD16_04835	276	1386	1342	0.2	1.1	0.860
928	CD16_04840	CD16_04840	753	1173	1150	0.1	1.1	0.840
929	CD16_04845	CD16_04845	243	46	266	-2.3	-5.0	0.160
930	CD16_04850	CD16_04850	216	0	0	NaN	NaN	NaN
931	CD16_04855	CD16_04855	1152	413	190	1.3	2.4	0.070
932	CD16_04860	CD16_04860	678	965	861	0.3	1.2	0.700
933	CD16_04865	CD16_04865	2109	471	406	0.4	1.3	0.490
934	murB	CD16_04875	1017	112	64	0.9	1.9	0.550
935	CD16_04880	CD16_04880	1425	1040	244	2.2	4.7	0.000
936	murG	CD16_04885	1110	402	455	-0.1	-1.0	0.950
937	CD16_04890	CD16_04890	1158	491	152	1.8	3.6	0.006
938	murD	CD16_04895	1407	408	594	-0.4	-1.3	0.450
939	CD16_04900	CD16_04900	1101	368	160	1.3	2.5	0.110
940	murF	CD16_04905	1419	184	232	-0.2	-1.1	0.830
941	CD16_04910	CD16_04910	1494	492	324	0.8	1.7	0.130
942	CD16_04915	CD16_04915	1797	215	168	0.5	1.4	0.590
943	CD16_04920	CD16_04920	378	558	579	0.1	1.0	0.950

944	rsmH	CD16_04925	1026	303	573	-0.8	-1.7	0.380
945	mraZ	CD16_04930	438	365	598	-0.6	-1.5	0.580
946	CD16_04935	CD16_04935	1629	558	162	1.9	3.8	0.001
947	CD16_04940	CD16_04940	1197	436	675	-0.5	-1.4	0.480
948	CD16_04945	CD16_04945	261	477	82	2.6	6.0	0.190
949	CD16_04950	CD16_04950	1263	551	902	-0.6	-1.5	0.190
950	CD16_04955	CD16_04955	897	461	267	0.9	1.8	0.250
951	CD16_04960	CD16_04960	825	172	350	-0.8	-1.8	0.420
952	CD16_04965	CD16_04965	681	338	349	0.1	1.0	0.960
953	CD16_04970	CD16_04970	792	358	249	0.7	1.6	0.450
954	CD16_04975	CD16_04975	1251	314	330	0.1	1.0	0.950
955	yajC	CD16_04980	330	989	1188	-0.1	-1.1	0.880
956	CD16_04985	CD16_04985	1476	839	798	0.2	1.1	0.660
957	lysS	CD16_04990	1509	414	190	1.3	2.4	0.050
958	CD16_04995	CD16_04995	258	76	0	4.3	19.2	0.690
959	rlmN	CD16_05000	1155	502	659	-0.3	-1.2	0.680
960	CD16_05005	CD16_05005	558	1201	706	0.9	1.9	0.160
961	CD16_05010	CD16_05010	669	284	296	0.1	1.1	0.950
962	CD16_05015	CD16_05015	1833	204	180	0.3	1.3	0.720
963	CD16_05020	CD16_05020	306	398	864	-1.0	-2.0	0.260
964	CD16_05025	CD16_05025	180	206	120	0.9	1.9	0.720
965	CD16_05030	CD16_05030	258	50	0	3.7	13.1	0.720
966	CD16_05035	CD16_05035	678	9112	3320	1.6	3.0	0.000
967	CD16_05040	CD16_05040	312	21	69	-1.5	-2.8	0.620
968	CD16_05045	CD16_05045	1137	473	807	-0.6	-1.6	0.150
969	CD16_05050	CD16_05050	453	121	289	-1.1	-2.1	0.390
970	CD16_05070	CD16_05070	717	228	180	0.5	1.4	0.710
971	CD16_05075	CD16_05075	480	34	91	-1.3	-2.5	0.560
972	CD16_05080	CD16_05080	1023	130	148	-0.1	-1.1	0.940
973	CD16_05085	CD16_05085	252	135	171	-0.2	-1.2	0.930
974	CD16_05090	CD16_05090	345	120	62	1.1	2.1	0.670
975	CD16_05095	CD16_05095	588	460	149	1.7	3.3	0.110
976	CD16_05100	CD16_05100	537	420	525	-0.2	-1.2	0.840
977	CD16_05105	CD16_05105	174	124	253	-0.9	-1.8	0.710
978	CD16_05110	CD16_05110	995	259	350	-0.3	-1.2	0.700
979	CD16_05115	CD16_05115	591	92	405	-2.0	-4.0	0.020
980	CD16_05120	CD16_05120	249	96	90	0.3	1.3	0.910
981	CD16_05735	CD16_05735	231	7	0	1.4	2.6	0.910
982	CD16_05125	CD16_05125	1053	107	512	-2.1	-4.4	0.030
983	CD16_05130	CD16_05130	1368	184	220	-0.2	-1.1	0.860
984	CD16_05740	CD16_05740	369	250	238	0.2	1.1	0.920
985	CD16_05135	CD16_05135	252	3218	3035	0.2	1.2	0.660
986	CD16_05745	CD16_05745	1449	313	362	-0.1	-1.1	0.920
987	CD16_05145	CD16_05145	498	134	389	-1.4	-2.7	0.280

988	CD16_05150	CD16_05150	198	86	222	-1.2	-2.3	0.590
989	CD16_05155	CD16_05155	465	3259	3983	-0.2	-1.1	0.680
990	CD16_05160	CD16_05160	258	2995	4478	-0.4	-1.4	0.250
991	CD16_05165	CD16_05165	1896	2974	2757	0.2	1.2	0.150
992	CD16_05170	CD16_05170	204	527	0	6.7	102.1	0.500
993	CD16_05175	CD16_05175	738	279	208	0.6	1.5	0.610
994	CD16_05180	CD16_05180	1374	235	236	0.1	1.1	0.920
995	CD16_05185	CD16_05185	441	2101	4754	-1.1	-2.1	0.000
996	CD16_05190	CD16_05190	645	430	892	-0.9	-1.9	0.270
997	CD16_05750	CD16_05750	150	0	0	NaN	NaN	NaN
998	pdxH	CD16_05195	606	279	397	-0.4	-1.3	0.690
999	CD16_05200	CD16_05200	855	2598	2898	0.0	-1.0	0.910
1000	dusA	CD16_05205	1053	595	769	-0.3	-1.2	0.630
1001	ulaA	CD16_05210	1386	1015	1289	-0.2	-1.2	0.540
1002	phrB	CD16_05215	1452	197	121	0.9	1.8	0.350
1003	CD16_05220	CD16_05220	1263	1221	637	1.1	2.1	0.002
1004	ligA	CD16_05225	2196	207	80	1.5	2.9	0.030
1005	recN	CD16_05230	1668	308	482	-0.5	-1.4	0.490
1006	bamD	CD16_05235	816	2188	2976	-0.3	-1.3	0.180
1007	CD16_05240	CD16_05240	891	316	391	-0.2	-1.1	0.810
1008	ftsZ	CD16_05245	1509	386	158	1.4	2.7	0.020
1009	ftsA	CD16_05250	1323	403	185	1.3	2.4	0.070
1010	CD16_05255	CD16_05255	915	303	216	0.6	1.6	0.450
1011	CD16_05260	CD16_05260	918	542	190	1.6	3.1	0.020
1012	CD16_05265	CD16_05265	216	53	0	3.5	11.6	0.730
1013	CD16_05270	CD16_05270	258	217	838	-1.8	-3.6	0.060
1014	CD16_05275	CD16_05275	285	268	230	0.4	1.3	0.840
1015	CD16_05280	CD16_05280	285	712	1620	-1.0	-2.0	0.070
1016	CD16_05285	CD16_05285	237	25	91	-1.7	-3.3	0.590
1017	CD16_05290	CD16_05290	1104	179	353	-0.9	-1.8	0.270
1018	CD16_05295	CD16_05295	558	90	0	5.6	48.3	0.590
1019	CD16_05300	CD16_05300	759	208	229	0.0	-1.0	0.980
1020	CD16_05305	CD16_05305	702	62	61	0.1	1.1	0.960
1021	CD16_05310	CD16_05310	498	299	263	0.4	1.3	0.770
1022	CD16_05315	CD16_05315	597	84	0	5.5	46.3	0.600
1023	CD16_05320	CD16_05320	2028	24	42	-0.7	-1.6	0.710
1024	CD16_05325	CD16_05325	297	72	0	4.5	22.2	0.670
1025	CD16_05330	CD16_05330	1383	123	62	1.1	2.1	0.480
1026	CD16_05335	CD16_05335	360	96	60	0.8	1.7	0.760
1027	CD16_05340	CD16_05340	573	93	155	-0.6	-1.5	0.730
1028	CD16_05345	CD16_05345	414	2481	3007	-0.2	-1.1	0.880
1029	CD16_05350	CD16_05350	580	8744	20510	-1.1	-2.2	0.000
1030	CD16_05355	CD16_05355	474	974	1112	-0.1	-1.0	0.930
1031	CD16_05360	CD16_05360	273	236	0	5.9	60.5	0.570

1032	CD16_05365	CD16_05365	945	774	736	0.2	1.2	0.710
1033	CD16_05370	CD16_05370	477	133	135	0.1	1.1	0.960
1034	CD16_05375	CD16_05375	273	169	798	-2.1	-4.2	0.060
1035	CD16_05755	CD16_05755	1185	294	257	0.3	1.2	0.710
1036	CD16_05385	CD16_05385	2430	242	241	0.1	1.1	0.870
1037	CD16_05390	CD16_05390	1224	147	160	0.0	-1.0	0.990
1038	CD16_05395	CD16_05395	2262	283	272	0.2	1.1	0.820
1039	CD16_05400	CD16_05400	342	670	504	0.5	1.4	0.660
1040	CD16_05405	CD16_05405	1725	645	1235	-0.8	-1.8	0.003
1041	CD16_05410	CD16_05410	1773	1352	2238	-0.6	-1.5	0.001
1042	CD16_05415	CD16_05415	519	234	249	0.1	1.0	0.970
1043	CD16_05420	CD16_05420	1032	371	2270	-2.5	-5.6	0.000
1044	CD16_05425	CD16_05425	741	224	2483	-3.3	-10.0	0.000
1045	CD16_05430	CD16_05430	273	20	79	-1.5	-2.8	0.610
1046	CD16_05435	CD16_05435	1620	217	1700	-2.8	-7.2	0.000
1047	CD16_05440	CD16_05440	1551	851	3053	-1.7	-3.3	0.000
1048	CD16_05445	CD16_05445	489	703	666	0.2	1.1	0.850
1049	CD16_05450	CD16_05450	546	911	1270	-0.4	-1.3	0.610
1050	CD16_05455	CD16_05455	222	49	585	-3.3	-10.0	0.030
1051	CD16_05460	CD16_05460	393	2204	1493	0.7	1.6	0.120
1052	CD16_05465	CD16_05465	207	41	0	3.2	8.9	0.770
1053	CD16_05470	CD16_05470	243	47	0	3.7	12.8	0.720
1054	CD16_05475	CD16_05475	333	48	393	-2.9	-7.5	0.020
1055	CD16_05485	CD16_05485	345	2715	6130	-1.1	-2.1	0.000
1056	CD16_05490	CD16_05490	2373	275	351	-0.2	-1.2	0.680
1057	CD16_05495	CD16_05495	390	141	55	1.4	2.7	0.610
1058	CD16_05500	CD16_05500	207	19	0	2.4	5.4	0.830
1059	CD16_05505	CD16_05505	270	38	80	-1.0	-2.0	0.730
1060	CD16_05510	CD16_05510	372	121	406	-1.6	-3.1	0.190
1061	CD16_05515	CD16_05515	324	37	0	3.6	12.2	0.730
1062	CD16_05520	CD16_05520	1167	641	1515	-1.1	-2.2	0.020
1063	CD16_05525	CD16_05525	789	63	166	-1.2	-2.3	0.280
1064	CD16_05530	CD16_05530	651	250	405	-0.6	-1.5	0.590
1065	CD16_05535	CD16_05535	2028	150	42	1.9	3.8	0.050
1066	CD16_05540	CD16_05540	312	53	0	4.0	16.1	0.710
1067	CD16_05545	CD16_05545	675	23	0	4.0	15.5	0.710
	Average			875	931			

*TPM, Transcripts per kilobase million. FDR, false discovery rate.

Table S2. Differentially expressed genes of "*Candidatus* Liberibacter asiaticus" between leaf midribs HiSeq data and fruit pith HiSeq data obtained by CLC Genomic Workbench 20.0. *Gene locus tag referenced to genome of CLas A4 strain (CP010804.2)

No.	Locus_ tag	Name	Log_2 fold change	Fold change	FDR*	Product
1	CD16_03055	CD16_03055	4.55	23.4	0.0100	L,D-transpeptidase family protein
2	CD16_02960	CD16_02960	3.92	15.1	0.0001	DNA translocase FtsK
3	CD16_04805	CD16_04805	3.86	14.5	0.0000	hypothetical protein
4	CD16_05730	CD16_05730	3.29	9.8	0.0000	hypothetical protein
5	CD16_04815	CD16_04815	3.26	9.6	0.0000	hypothetical protein
6	CD16_01910	secB	3.22	9.3	0.0100	protein-export chaperone SecB
7	CD16_03130	CD16_03130	3.18	9.1	0.0100	cysteine-tRNA ligase
8	CD16_04810	CD16_04810	2.90	7.5	0.0000	hypothetical protein
9	CD16_03885	CD16_03885	2.88	7.4	0.0012	DUF1153 domain-containing protein
10	CD16_03935	CD16_03935	2.87	7.3	0.0300	TrmJ/YjtD family RNA methyltransferase
11	CD16_04615	sdhC	2.86	7.3	0.0300	succinate dehydrogenase, cytochrome b556 subunit
12	CD16_02455	ftsY	2.75	6.7	0.0500	signal recognition particle-docking protein FtsY
13	CD16_04475	CD16_04475	2.66	6.3	0.0005	orotate phosphoribosyltransferase
14	CD16_00875	topA	2.54	5.8	0.0000	type I DNA topoisomerase
15	CD16_00590	rplE	2.52	5.7	0.0024	50S ribosomal protein L5
16	CD16_02070	rpsO	2.48	5.6	0.0037	30S ribosomal protein S15
17	CD16_03670	CD16_03670	2.47	5.5	0.0000	NADH-quinone oxidoreductase subunit B
18	CD16_02590	surE	2.30	4.9	0.0017	5'/3'-nucleotidase SurE
19	CD16_04880	CD16_04880	2.22	4.7	0.0000	UDP-N-acetylmuramate--L-alanine ligase
20	CD16_01055	CD16_01055	2.20	4.6	0.02	Choline ABC transporter, ATP-binding protein
21	CD16_03845	CD16_03845	2.12	4.4	2.7E-11	PilZ domain-containing protein
22	CD16_00575	rplF	2.11	4.3	0.0300	50S ribosomal protein L6
23	CD16_03485	plsX	2.07	4.2	0.0001	phosphate acyltransferase PlsX
24	CD16_00685	accC	2.02	4.1	0.0000	acetyl-CoA carboxylase biotin carboxylase subunit
25	CD16_02910	atpD	2.01	4.0	0.0000	F0F1 ATP synthase subunit beta
26	CD16_04580	CD16_04580	1.98	3.9	0.0001	hypothetical protein
27	CD16_00680	CD16_00680	1.95	3.9	0.0100	biotin/lipoyl-binding protein
28	CD16_03585	CD16_03585	1.94	3.8	0.0400	Type I restriction endonuclease subunit S
29	CD16_04935	CD16_04935	1.92	3.8	0.0005	alpha-D-glucose phosphate-specific phosphoglucomutase
30	CD16_05535	CD16_05535	1.92	3.8	0.0500	DNA polymerase
31	CD16_02510	CD16_02510	1.90	3.7	0.0300	hypothetical protein
32	CD16_02870	ubiB	1.90	3.7	0.0300	2-polyprenylphenol 6-hydroxylase
33	CD16_00990	secA	1.83	3.6	0.0000	preprotein translocase subunit SecA
34	CD16_04890	CD16_04890	1.83	3.6	0.0059	cell division protein FtsW
35	CD16_03230	ruvB	1.81	3.5	0.0400	Holliday junction branch migration DNA helicase RuvB
36	CD16_04820	CD16_04820	1.80	3.5	0.0100	hypothetical protein
37	CD16_03985	CD16_03985	1.77	3.4	0.0000	oligoendopeptidase F
38	CD16_04655	sucD	1.75	3.4	0.0015	succinate--CoA ligase subunit alpha
39	CD16_03470	CD16_03470	1.74	3.3	0.0000	ribonucleoside-diphosphate reductase subunit alpha
40	CD16_04560	CD16_04560	1.70	3.2	0.0000	chemotaxis protein
41	CD16_02710	gap	1.70	3.3	0.0004	type I glyceraldehyde-3-phosphate dehydrogenase
42	CD16_02500	CD16_02500	1.67	3.2	0.0013	sensor histidine kinase

43	CD16_00495	CD16_00495	1.65	3.1	0.0000	DUF4011 domain-containing protein
44	CD16_04240	CD16_04240	1.63	3.1	0.0002	Bcr/CflA family efflux MFS transporter
45	CD16_05260	CD16_05260	1.62	3.1	0.0200	D-alanine--D-alanine ligase
46	CD16_05035	CD16_05035	1.58	3.0	0.0000	hypothetical protein
47	CD16_00580	rpsH	1.55	2.9	0.0200	30S ribosomal protein S8
48	CD16_02645	CD16_02645	1.53	2.9	0.0021	phosphopyruvate hydratase
49	CD16_03910	map	1.52	2.9	0.0200	type I methionyl aminopeptidase
50	CD16_05225	ligA	1.51	2.9	0.0300	NAD-dependent DNA ligase LigA
51	CD16_01110	CD16_01110	1.50	2.8	0.0300	winged helix DNA-binding protein
52	CD16_04035	secD	1.49	2.8	0.0000	protein translocase subunit SecD
53	CD16_00470	CD16_00470	1.49	2.8	0.0008	DUF2336 domain-containing protein
54	CD16_02855	rpsT	1.48	2.8	0.0000	30S ribosomal protein S20
55	CD16_04000	CD16_04000	1.45	2.7	0.0000	glycosyltransferase
56	CD16_02000	CD16_02000	1.44	2.7	0.0037	cell division protein FtsK
57	CD16_01495	CD16_01495	1.43	2.7	0.0100	NifU family protein
58	CD16_02330	CD16_02330	1.42	2.7	0.0000	Flp family type IVb pilin
59	CD16_01550	CD16_01550	1.42	2.7	0.0047	glycosyltransferase
60	CD16_05245	ftsZ	1.41	2.7	0.0200	cell division protein FtsZ
61	CD16_02625	CD16_02625	1.40	2.6	0.0019	pyruvate dehydrogenase complex dihydrolipoamide acetyltransferase
62	CD16_03975	CD16_03975	1.40	2.6	0.0021	cold-shock protein
63	CD16_00550	secY	1.40	2.7	0.0200	preprotein translocase subunit SecY
64	CD16_00620	rpsC	1.39	2.6	0.0027	30S ribosomal protein S3
65	CD16_01860	CD16_01860	1.38	2.6	0.0076	phosphoenolpyruvate carboxykinase
66	CD16_02035	CD16_02035	1.38	2.6	0.0500	aminopeptidase
67	CD16_01545	greA	1.36	2.6	0.0012	transcription elongation factor GreA
68	CD16_02905	CD16_02905	1.36	2.6	0.0500	F0F1 ATP synthase subunit gamma
69	CD16_00705	CD16_00705	1.33	2.5	0.0015	ATP-dependent Clp protease proteolytic subunit
70	CD16_00805	CD16_00805	1.32	2.5	0.0300	hypothetical protein
71	CD16_00210	CD16_00210	1.31	2.5	0.0019	ABC transporter permease subunit
72	CD16_03695	CD16_03695	1.30	2.5	0.0057	NADH-quinone oxidoreductase subunit G
73	CD16_01125	CD16_01125	1.30	2.5	0.0100	citrate synthase
74	CD16_02595	serS	1.27	2.4	0.0000	serine--tRNA ligase
75	CD16_01735	CD16_01735	1.27	2.4	0.0500	response regulator
76	CD16_04990	lysS	1.26	2.4	0.0500	lysine--tRNA ligase
77	CD16_00930	CD16_00930	1.25	2.4	0.0000	hypothetical protein
78	CD16_01205	lysA	1.23	2.4	0.0067	diaminopimelate decarboxylase
79	CD16_00525	rplQ	1.23	2.3	0.0400	50S ribosomal protein L17
80	CD16_01170	CD16_01170	1.23	2.4	0.0400	ferredoxin--NADP reductase
81	CD16_01780	bioB	1.22	2.3	0.0005	biotin synthase BioB
82	CD16_00065	rpoC	1.21	2.3	0.0000	DNA-directed RNA polymerase subunit beta'
83	CD16_00070	rpoB	1.21	2.3	0.0000	DNA-directed RNA polymerase subunit beta
84	CD16_03310	fabG	1.20	2.3	0.0100	3-oxoacyl-[acyl-carrier-protein] reductase
85	CD16_01665	CD16_01665	1.19	2.3	0.0000	peptidoglycan-binding protein
86	CD16_00815	rpsI	1.18	2.3	0.0002	30S ribosomal protein S9
87	CD16_01755	CD16_01755	1.18	2.3	0.0060	2'-deoxycytidine 5'-triphosphate deaminase
88	CD16_01785	CD16_01785	1.17	2.2	0.0093	aminotransferase class I/II-fold pyridoxal phosphate-dependent enzyme
89	CD16_04220	CD16_04220	1.16	2.2	0.0200	DUF882 domain-containing protein

90	CD16_00480	ychF	1.16	2.2	0.0500	redox-regulated ATPase YchF
91	CD16_02395	CD16_02395	1.15	2.2	0.0200	Pilus assembly protein TadC
92	CD16_00645	rplD	1.14	2.2	0.0100	50S ribosomal protein L4
93	CD16_02675	CD16_02675	1.09	2.1	0.0068	NAD-glutamate dehydrogenase
94	CD16_01705	infA	1.07	2.1	0.0004	translation initiation factor IF-1
95	CD16_02990	CD16_02990	1.07	2.1	0.0200	aminotransferase class I/II-fold pyridoxal phosphate-dependent enzyme
96	CD16_00510	typA	1.06	2.1	0.0010	translational GTPase TypA
97	CD16_03730	nuoN	1.06	2.1	0.0200	NADH-quinone oxidoreductase subunit NuoN
98	CD16_02255	CD16_02255	1.05	2.1	0.0014	PBP1A family penicillin-binding protein
99	CD16_05220	CD16_05220	1.05	2.1	0.0015	cation:dicarboxylase symporter family transporter
100	CD16_02060	infB	1.05	2.1	0.0200	translation initiation factor IF-2
101	CD16_01660	CD16_01660	1.04	2.1	0.0200	SDR family oxidoreductase
102	CD16_01275	CD16_01275	-1.01	-2.0	0.0001	ATP-binding cassette domain-containing protein
103	CD16_04185	CD16_04185	-1.01	-2.0	0.0035	hypothetical protein
104	CD16_03005	uvrC	-1.01	-2.0	0.0300	excinuclease ABC subunit UvrC
105	CD16_00710	ettA	-1.01	-2.0	0.0400	energy-dependent translational throttle protein EttA
106	CD16_02475	pstC	-1.03	-2.1	0.0200	phosphate ABC transporter permease subunit PstC
107	CD16_02285	CD16_02285	-1.04	-2.1	0.0300	hypothetical protein
108	CD16_05485	CD16_05485	-1.05	-2.1	0.0000	hypothetical protein
109	CD16_02535	CD16_02535	-1.06	-2.1	0.0030	LuxR family transcriptional regulator
110	CD16_03710	CD16_03710	-1.06	-2.1	0.0500	NADH-quinone oxidoreductase subunit J
111	CD16_05185	CD16_05185	-1.07	-2.1	0.0000	GNAT family N-acetyltransferase
112	CD16_04235	CD16_04235	-1.07	-2.1	0.0200	hypothetical protein
113	CD16_04210	serB	-1.08	-2.1	0.0300	phosphoserine phosphatase SerB
114	CD16_00235	CD16_00235	-1.09	-2.1	0.0200	hypothetical protein
115	CD16_05350	CD16_05350	-1.10	-2.2	0.0000	hypothetical protein
116	CD16_04505	CD16_04505	-1.11	-2.2	0.0100	GHMP kinase
117	CD16_05520	CD16_05520	-1.11	-2.2	0.0200	DUF2800 domain-containing protein
118	CD16_00835	CD16_00835	-1.15	-2.2	0.0400	peptide deformylase
119	CD16_03185	CD16_03185	-1.20	-2.3	0.0300	thiamine ABC transporter substrate binding subunit
120	CD16_03000	pgsA	-1.20	-2.3	0.0500	CDP-diacylglycerol-glycerol-3-phosphate 3-phosphatidyltransferase
121	CD16_00435	CD16_00435	-1.21	-2.3	0.0057	DNA polymerase III subunit delta'
122	CD16_02880	CD16_02880	-1.24	-2.4	0.0000	Hsp20 family protein
123	CD16_01930	CD16_01930	-1.25	-2.4	0.0001	septum formation inhibitor Maf
124	CD16_02165	recF	-1.25	-2.4	0.0300	DNA replication/repair protein RecF
125	CD16_02280	sppA	-1.26	-2.4	0.0000	signal peptide peptidase SppA
126	CD16_03285	CD16_03285	-1.26	-2.4	0.0073	hypothetical protein
127	CD16_01000	rpsF	-1.33	-2.5	0.0000	30S ribosomal protein S6
128	CD16_00155	CD16_00155	-1.34	-2.5	0.0001	hypothetical protein
129	CD16_02940	CD16_02940	-1.34	-2.5	0.0022	RluA family pseudouridine synthase
130	CD16_00405	CD16_00405	-1.37	-2.6	0.0400	hypothetical protein
131	CD16_01450	CD16_01450	-1.42	-2.7	0.0000	tetratricopeptide repeat protein
132	CD16_03300	CD16_03300	-1.42	-2.7	0.0000	metal ABC transporter substrate-binding protein

133	CD16_02010	CD16_02010	-1.42	-2.7	0.0200	endonuclease/exonuclease/phosphatase family protein
134	CD16_02835	CD16_02835	-1.44	-2.7	0.0100	ribonuclease PH
135	CD16_00375	CD16_00375	-1.45	-2.7	0.0002	hypothetical protein
136	CD16_02975	CD16_02975	-1.46	-2.8	0.0057	23S rRNA (guanosine(2251)-2'-O)-methyltransferase RlmB
137	CD16_03365	CD16_03365	-1.47	-2.8	0.0200	flagellar hook-basal body complex protein
138	CD16_04430	CD16_04430	-1.50	-2.8	0.0000	endonuclease/exonuclease/phosphatase family protein
139	CD16_01770	CD16_01770	-1.50	-2.8	0.0014	YihA family ribosome biogenesis GTP-binding protein
140	CD16_04360	CD16_04360	-1.52	-2.9	0.0040	GNAT family N-acetyltransferase
141	CD16_03175	CD16_03175	-1.53	-2.9	0.0022	ABC transporter substrate-binding protein
142	CD16_03865	CD16_03865	-1.54	-2.9	0.0030	histidine phosphotransferase
143	CD16_03160	CD16_03160	-1.56	-3.0	0.0040	hypothetical protein
144	CD16_01575	CD16_01575	-1.58	-3.0	0.0500	hypothetical protein
145	CD16_02570	motA	-1.59	-3.0	0.0010	flagellar motor stator protein MotA
146	CD16_03420	flhA	-1.62	-3.1	0.0091	flagellar biosynthesis protein FlhA
147	CD16_03235	CD16_03235	-1.65	-3.1	0.0012	biopolymer transporter ExbB
148	CD16_05440	CD16_05440	-1.71	-3.3	0.0000	terminase
149	CD16_04425	CD16_04425	-1.72	-3.3	0.0085	hypothetical protein
150	CD16_01850	hslV	-1.73	-3.3	0.0073	ATP-dependent protease subunit HslV
151	CD16_04740	CD16_04740	-1.77	-3.4	0.0049	hypothetical protein
152	CD16_03350	CD16_03350	-1.84	-3.6	0.0100	chemotaxis protein
153	CD16_02515	CD16_02515	-1.98	-4.0	0.0003	D-alanyl-D-alanine carboxypeptidase
154	CD16_05115	CD16_05115	-2.00	-4.0	0.0200	hypothetical protein
155	CD16_03120	CD16_03120	-2.03	-4.1	0.0000	EamA family transporter
156	CD16_05125	CD16_05125	-2.12	-4.4	0.0300	hypothetical protein
157	CD16_00965	CD16_00965	-2.15	-4.4	0.0000	methyltransferase domain-containing protein
158	CD16_00025	CD16_00025	-2.38	-5.2	0.0300	tyrosine-type recombinase/integrase
159	CD16_05420	CD16_05420	-2.48	-5.6	0.0000	hypothetical protein
160	CD16_04315	CD16_04315	-2.63	-6.2	0.0000	LysE family translocator
161	CD16_05435	CD16_05435	-2.84	-7.2	0.0000	phage tail protein
162	CD16_03335	CD16_03335	-2.87	-7.3	0.0000	flagellin
163	CD16_05475	CD16_05475	-2.91	-7.5	0.0200	hypothetical protein
164	CD16_03440	CD16_03440	-2.93	-7.6	0.0100	hypothetical protein
165	CD16_01255	flgB	-3.08	-8.5	0.0000	flagellar basal body rod protein FlgB
166	CD16_05425	CD16_05425	-3.32	-10.0	0.0000	hypothetical protein
167	CD16_05455	CD16_05455	-3.32	-10.0	0.0300	hypothetical protein
168	CD16_04420	CD16_04420	-3.67	-12.7	0.0023	hypothetical protein
169	CD16_05555	CD16_05555	-3.74	-13.3	0.0042	hypothetical protein

FDR, false discovery rate.
